# Simulating cell-free chromatin using preclinical cancer models for liquid biopsy applications

**DOI:** 10.1016/j.isci.2025.114113

**Published:** 2025-11-22

**Authors:** Sasha C. Main, Steven D. De Michino, Lucas Penny, Aleem Aamir, Tina Keshavarzian, Benjamin H. Lok, Robert Kridel, David W. Cescon, Michael M. Hoffman, Mathieu Lupien, Scott V. Bratman

**Affiliations:** 1 Princess Margaret Cancer Centre, University Health Network, Toronto, ON M5G 2C1, Canada; 2 Department of Medical Biophysics, University of Toronto, Toronto, ON M5G 1L7, Canada; 3 Division of Medical Oncology and Hematology, Department of Medicine, University of Toronto, Toronto, ON M5S 1A8, Canada; 4 Institute of Medical Sciences, University of Toronto, Toronto, ON M5S 1A8, Canada; 5 Department of Computer Science, University of Toronto, Toronto, ON M5S 2E4, Canada; 6 Vector Institute for Artificial Intelligence, Toronto, ON M5G 1M1, Canada; 7 Ontario Institute for Cancer Research, Toronto, ON M5G 0A3, Canada; 8 Department of Radiation Oncology, University of Toronto, Toronto, ON M5T 1P5, Canada

**Keywords:** medical biotechnology, genomics, chromosome organization

## Abstract

Cell-free DNA circulates in blood bound to nucleosomes, forming cell-free chromatin (cfChromatin) that retains epigenetic features, including nucleosome positioning and histone modifications. cfChromatin provides a rich source of cancer biomarkers; however, low abundance of tumor-derived cfChromatin and limited availability of clinical samples pose challenges for liquid biopsy research. To address this, we developed a framework to simulate cfChromatin nucleosomal distributions using nuclease-treated conditioned media from tissue cultures. Whole-genome sequencing confirmed that inferred nucleosome positioning reflected cell-type-specific gene expression and chromatin accessibility patterns, and comparisons with plasma cfChromatin from xenografted mice revealed concordant nucleosome profiles. Notably, simulated cfChromatin displayed stronger tumor-specific nucleosome profiles than patient plasma, where hematopoietic-derived cfChromatin dilutes signal. We further leveraged simulated cfChromatin to advance cell-free chromatin immunoprecipitation and sequencing methods, identifying repressive and bivalent chromatin domains predictive of transcriptional activity. Altogether, our results demonstrate the utility of simulated cfChromatin as a scalable preclinical tool for liquid biopsy research.

## Highlights

• Nuclease treatment of media simulates cfChromatin nucleosomal distributions
• Simulated cfChromatin retains undiluted cancer-associated chromatin configurations
• This platform advanced methods for cfChIP-seq targeting both H3K4me3 and H3K27me3
• cfChIP-seq of both marks revealed bivalency and improved gene expression prediction

## Graphical abstract

**Figure:**
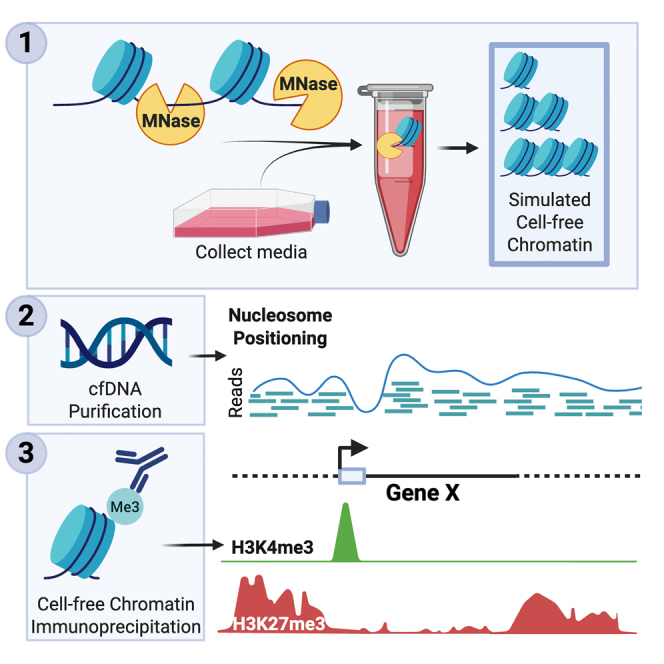

## Introduction

In the past decade, analysis of cancer-specific analytes through a simple blood test, known as liquid biopsy, has emerged as a practice-changing approach for minimally invasive detection of cancer biomarkers.^1^ Numerous liquid biopsy analytes have been explored in the context of cancer, most notably circulating tumor cells and cell-free DNA (cfDNA).^1^ cfDNA is released into the bloodstream through various mechanisms—predominantly apoptosis and necrosis—and is digested into short nucleosomal fragments by nucleases.^2^ Most cfDNA originates from hematopoietic cells,^3^ but in individuals with cancer, a portion of the cfDNA pool consists of tumor-derived fragments that retain the tumor’s genetic and epigenetic alterations. Analysis of tumor-specific cfDNA biomarkers through liquid biopsy provides advantages compared to traditional tissue biopsies, such as overcoming spatial limitations, allowing easily repeatable measurements over time,^4^ and increasing cost efficiency in certain clinical contexts.^5^

The characterization of tumor-specific genetic alterations within cfDNA has resulted in promising biomarkers for cancer management.^6^^,^^7^ However, genetic mutations do not reflect the full spectrum of cancer molecular or cellular phenotypes and provide limited information about tumor biology under the influence of anti-cancer therapy.^2^^,^^8^ Widespread epigenetic aberrations in cancer, such as changes in DNA methylation, histone modifications, and nucleosome positioning, influence transcriptional activity and cellular phenotypes. The pivotal role of such alterations has motivated the development of new classes of liquid biopsy biomarkers utilizing cell-free chromatin (cfChromatin).^9^

Accordingly, emerging methods now enable the profiling of epigenetic features in cfChromatin.^2^^,^^9^^,^^10^^,^^11^^,^^12^^,^^13^ For instance, plasma cfChromatin is nucleosome bound and protected from nuclease digestion, so it can be leveraged for inferring nucleosome positioning through whole-genome sequencing (WGS).^14^^,^^15^^,^^16^ This approach resembles the traditional use of nuclease digestion patterns to deduce chromatin structure in cells, such as sequencing protected fragments from micrococcal nuclease (MNase) treatment.^17^^,^^18^^,^^19^^,^^20^^,^^21^^,^^22^ Nucleosome positioning inferred from plasma cfChromatin provides insight into chromatin structure and expression programs from the tissue of origin.^14^^,^^15^^,^^19^^,^^23^^,^^24^

Recent methodologic developments have expanded cfChromatin profiling to include histone post-translational modifications. Proof-of-concept studies have demonstrated that histone modifications linked to active (euchromatin) and repressive (heterochromatin) chromatin states can be detected within blood plasma.^12^^,^^25^^,^^26^^,^^27^ These histone modifications collectively mark and define chromatin states, enabling the identification of cell-type-specific regulatory programs and prediction of transcriptional activity.^28^ Notably, Sadeh and colleagues developed cell-free chromatin immunoprecipitation and sequencing (cfChIP-seq)^25^ to profile transcriptionally active chromatin. However, sequencing-based profiling of cfChromatin marked by repressive histone modifications, characteristic of heterochromatin, remains largely unexplored, despite its importance for understanding transcriptional and regulatory element repression, cell-of-origin classification, and mechanisms of therapy resistance in cancer.

A key limitation in liquid biopsy technical development and biomarker discovery is the heavy reliance on patient plasma samples.^29^ Patient samples are not only finite and precious but also challenging to use for assay development due to the variability in tumor-derived cfChromatin fractions and the difficulty in obtaining matched tumor tissue as a ground truth reference for benchmarking. For plasma cfChromatin, this can be particularly problematic due to the <0.1% abundance of tumor-derived material observed in many cancer contexts.^30^^,^^31^^,^^32^ Additionally, overlapping expression patterns of putative epigenetic biomarkers across tissue types further complicate efforts to identify robust, cancer-specific signatures.^3^^,^^4^ To mitigate these issues, previous work has focused on selecting samples with high tumor-derived cfChromatin fractions or by performing deeper sequencing.^14^^,^^19^^,^^23^ While this may be suitable for certain high-burden disease settings, complex biological factors that limit the shedding of cfChromatin from many tumors,^33^ combined with high cost, hamper broader utilization. Moreover, these approaches do not resolve the scarcity of matched tissue samples needed for validation, nor do they overcome reproducibility issues inherent to limited and heterogeneous samples, posing significant barriers to achieving sufficient material for assay development and biomarker discovery.

Xenograft plasma samples have also been used in the liquid biopsy field, as they enable near-pure cancer-derived cfChromatin analysis through bioinformatic subtraction of mouse reads.^16^^,^^17^^,^^29^ However, they are labor intensive, can require long engraftment periods, and often yield low amounts of cancer-derived cfChromatin from small plasma volumes,^34^ impeding large-scale assay development and biomarker discovery.

To address these challenges, there is a demand for models where cancer-derived cfChromatin is readily accessible and abundant, can be studied without the confounding effects of signal dilution, and permits simple matched cellular data generation. Preclinical cancer models grown in tissue culture release cfChromatin that is entirely cancer derived and easily accessible,^34^^,^^35^ providing an opportunity to accelerate epigenetic liquid biopsy biomarker discovery and assay development. However, the natural fragment length distribution of conditioned media cfChromatin contains mainly large fragments (600 bp to >10 kb), which do not reflect the nucleosomal distributions observed in plasma and are less amenable to downstream profiling techniques, particularly those involving immunoprecipitation and/or short-read sequencing.

Here, we present a framework using a nuclease treatment to simulate cfChromatin of nucleosomal distributions similar to plasma within conditioned media of preclinical cancer models. We find that simulated cfChromatin retains nuclear chromatin accessibility profiles and their established relationships with gene expression, while demonstrating concordance with cancer-derived plasma cfChromatin from xenograft models. Due to their purity, these profiles are accentuated in simulated cfChromatin across a variety of cancer types compared with patient plasma samples. We further leveraged simulated cfChromatin as a renewable and reproducible source of material for the development of cfChIP-seq targeting histone modifications associated with heterochromatin. Altogether, we demonstrate the versatility of simulated cfChromatin as a preclinical tool for liquid biopsy method development and epigenetic biomarker discovery for further interrogation in patient samples.

## Results

### Nuclease treatment of cell-line-conditioned media reproducibly generates nucleosomal distributions from cfChromatin

**Figure 1:**
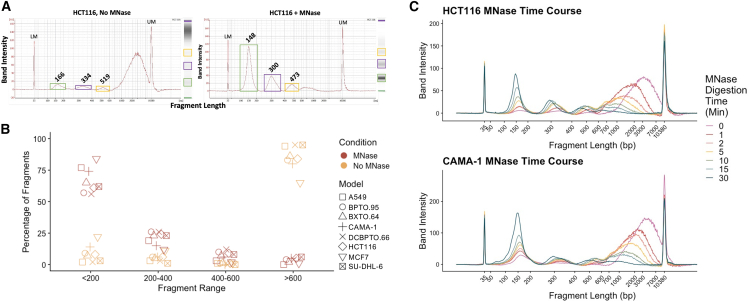
MNase treatment of media from preclinical models reproducibly generates nucleosomal distributions from cfChromatin (A) HCT116-conditioned media without (left) or with (right) MNase treatment shows enrichment for mono- and oligo-nucleosome-sized cfDNA fragments. LM, lower marker; UM, upper marker. (B) MNase treatment generates significantly different proportions of mono-, di-, and tri-cell-free nucleosomes compared to no nuclease treatment, reproducible across various 2D and organoid culture models (CAMA-1 and MCF7 = breast adenocarcinoma, HCT116 = colorectal carcinoma, A549 = lung adenocarcinoma, SU-DHL-6 = diffuse large B cell lymphoma, BPTO.95 and DCBPTO.66 = breast cancer patient-derived tumor organoids, BXTO.64 = breast cancer patient-derived xenograft-derived organoid). Paired *t* test for fragments (<200 bp, *p* = 1.53 × 10^−7^; 200–400 bp, *p* = 5.05 × 10^−4^; 400–600 bp, *p* = 1.44 × 10^−3^; >600 bp, *p* = 6.07 × 10^−8^). Results from (B) are extracted from the BioAnalyzer traces shown in Figure S1B. (C) HCT116 (top) and CAMA-1 (bottom) media samples were treated with a fixed concentration of MNase over a 0- to 30-min time course. The proportion of mononucleosome-sized fragments increases with digestion time, with a small fraction of mononucleosomes at earlier time points relative to later.

To overcome the limitations of current cfChromatin liquid biopsy discovery tools, we devised a framework for simulating cell-free nucleosomes using tissue culture-conditioned media (graphical abstract). First, we collected media from cultured HCT116 (colon cancer) cells during log-phase growth and measured cfChromatin topology by DNA fragment size analysis (Figure 1A). In stark contrast to the mono- and oligo-nucleosomal topology of cfChromatin present in human plasma,^14^ this native untreated cfChromatin contained predominantly DNA fragments larger than 600 bp in length (median ∼3,000 bp).

To generate cfChromatin with nucleosomal distributions more reflective of human plasma, we employed nucleases *in vitro* to digest HCT116-conditioned-media cfChromatin. We first performed digestion with MNase, as it is commonly used in “native” chromatin immunoprecipitation sequencing (ChIP-seq) experiments in place of sonication to avoid disturbing transcription factor association with DNA,^36^ in cleavage under targets and release using nuclease,^37^ and for mapping nucleosome occupancy.^17^^,^^18^^,^^19^^,^^20^^,^^21^^,^^22^ Following 30 min of digestion, we observed a shift from large fragments to smaller fragments corresponding to the size of mono-, di-, and trinucleosomes, with the median size of the mononucleosomal DNA fragments being 148 bp (Figure 1A). However, this desired size distribution was not fully maintained in culture media that was subjected to freeze-thaw (Figure S1A), indicating that fresh media is preferred to preserve nucleosome integrity.

Having demonstrated the feasibility of nuclease digestion of media cfChromatin with MNase, we next tested nucleases implicated in plasma cfDNA fragmentation, namely DNase1 and DNase1L3.^38^ After confirming the enzymatic activity of both nucleases with gDNA digestions (Figures S1B and S1C), we investigated their effects on media cfChromatin. Digestion with increasing concentrations of DNase1 resulted in progressive loss of larger fragments but did not substantially increase the abundance of mononucleosome-sized fragments (Figure S1D). Similarly, DNase1L3 digestion at the tested concentrations led to only minor increases in mononucleosome-sized fragments (100–200 bp range increased from 7% to 12%) (Figure S1D). Altogether, these findings indicate that while DNase1 and DNase1L3 were less effective in generating nucleosome-sized fragments, MNase treatment reliably simulates cfChromatin of nucleosomal distributions similar to plasma within conditioned media. We refer to this MNase-digested material as simulated cfChromatin.

### Simulated cfChromatin is robustly produced across tissue culture models

We next evaluated the generalizability of MNase treatment to produce nucleosome-sized simulated cfChromatin in other preclinical tissue culture models. We observed a significant difference in the fragment length distributions between MNase-treated and untreated conditions across 2D and 3D culture models (Figure 1B; Figure S1E). MNase-treated simulated cfChromatin consistently captured nucleosomal size distributions resembling those in human plasma, whereas untreated media cfChromatin was enriched for fragments greater than 600 bp. Recognizing the intrinsic variability in the amount of cfChromatin released among distinct models,^34^^,^^35^ we confirmed consistent nucleosome size distributions across a range of cfChromatin input quantities and volumes (Figure S1F).

Maintenance of the characteristic mononucleosomal size distribution within cfChromatin was strongly time dependent. While consistent results were observed after 30 min of MNase digestion, extended treatment for 4 or 24 h resulted in a reduction of median mononucleosomal DNA fragment size to 125 and 94 bp, respectively (Figure S1G). Conversely, shorter digestion times resulted in smaller proportions of mononucleosomes (Figure 1C; Figure S1H), with gradual shortening of the mononucleosome-associated fragments over time. Of note, CAMA-1 media contained more natural mononucleosomes before MNase digestion compared to HCT116 (Figure 1C), which is possibly related to differences in mechanisms of cell death or intracellular nuclease activity.^34^ Taken together, these results demonstrate that MNase digestion robustly produces nucleosome-sized simulated cfChromatin across diverse tissue culture models. The predictable fragment size distributions reflect the ability of MNase to shear unprotected DNA within media cfChromatin while maintaining the histone core complex, making this approach highly suitable for downstream profiling.

### Genome-wide simulated cfChromatin profiles reflect chromosomal variation

After demonstrating robust production of simulated cfChromatin, we sought to explore features revealed through its genomic profiling. We purified cfDNA from simulated cfChromatin across all models, including the CAMA-1 and HCT116 digestion time course samples, and conducted WGS (2.5× median coverage). This simulated cfChromatin sequencing data, which we term cell-free MNase-seq (cfMNase-seq), allowed us to investigate genome-wide nucleosome coverage and chromosomal features.

**Figure 2:**
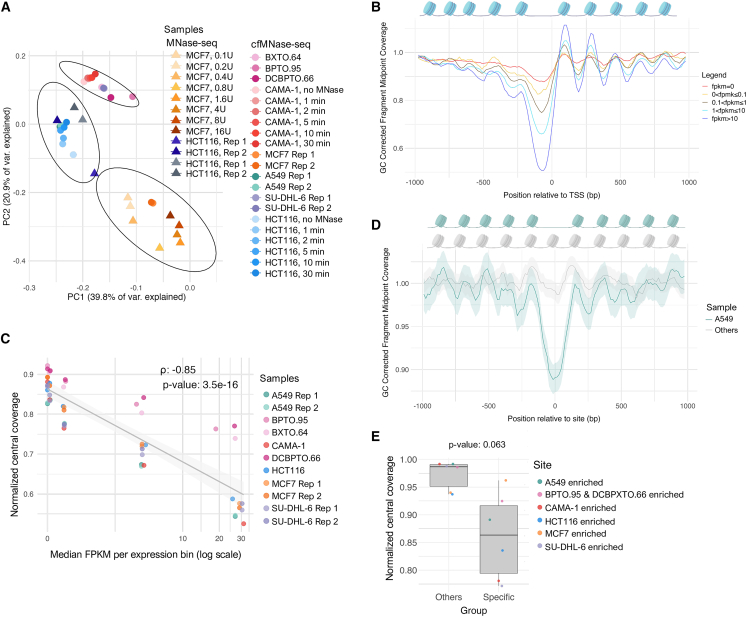
Simulated cfChromatin reflects chromosomal variation and nucleosome profiles associated with gene expression and chromatin accessibility patterns (A) PCA of cfMNase-seq samples across various sample types and MNase digestion times (*n* = 21) and samples from three external MNase-seq datasets for HCT116 (*n* = 4) and MCF7 (*n* = 8). The PCA was performed over 10,000 bp bins genome-wide. A visual representation of the 10,000 bp bin size is shown in Figure S2A. Clusters were identified using *k*-means clustering. A silhouette score was calculated to validate clustering; cluster 1 (bottom right) had an average silhouette score of 0.92, cluster 2 (middle left) 0.94, and cluster 3 (top middle) 0.96. (B) Analysis of the association between cfMNase-seq coverage and gene expression around the TSS. Composite cfMNase-seq coverage profiles at TSSs falling within five FPKM levels shown for CAMA-1 (30-min digestion). Coverage is shown as average GC-corrected fragment midpoint coverage. The complementary analysis for all other models, information on the FPKM subsets, and coverage profiles across MNase digestion times are shown in Figure S3. (C) Normalized central coverage metric from cfMNase-seq coverage profiles across different gene expression levels (Spearman correlation ρ = −0.85, *p* = 3.5 × 10^−16^), shown for all samples with a 30-min digestion time. (D) Composite cfMNase-seq coverage profiles (mean ±95% confidence interval) at 6,260 sites with enriched chromatin accessibility for A549, shown for A549 and the average of BPTO.95, CAMA-1, DCBPTO.66, HCT116, MCF7, and SU-DHL-6 grouped together (all 30-min digestions). The complementary results for other model-specific enriched open chromatin sites are shown in Figure S4D. (E) Normalized central coverage metric from cfMNase-seq coverage profiles across sites of enriched chromatin accessibility for each model (specific) compared to the average across the rest (others) (paired Wilcoxon signed-rank test *p* = 0.063).

To confirm that simulated cfChromatin reflects broad nuclear chromosomal variation, we compared all cfMNase-seq profiles with nuclear MNase-seq profiles for HCT116 from two independent studies and for MCF7 from one study that included eight different digestion levels.^39^^,^^40^ Principal-component analysis (PCA) of broad genome-wide nucleosome coverage (Figure S2A) revealed three distinct clusters with silhouette scores of 0.92, 0.94, and 0.96 (Figure 2A). All cfMNase-seq and nuclear MNase-seq profiles for HCT116 and MCF7 clustered together within their respective groups. Notably, the HCT116 cluster also included A549 cfMNase-seq samples. The remaining cluster comprised cfMNase-seq samples from CAMA-1 across all digestion times, breast cancer organoids, and SU-DHL-6. These results confirm that genome-wide nucleosome profiles captured by cfMNase-seq are similar to their corresponding nuclear MNase-seq signal, reflecting broad chromosomal features of the originating cell lines.

As plasma WGS can be used to measure cancer-specific chromosomal structural variants such as copy-number aberrations, we suspected that the genome-wide nucleosome coverage revealed by cfMNase-seq could also be used for this purpose. Therefore, we applied ichorCNA, commonly used for plasma WGS copy-number analysis,^41^ to all cfMNase-seq profiles (Figures S2B–S2F). Distinct copy-number profiles were obtained across models. For instance, HCT116 copy-number gains were evident for chromosomes 8, 10, 16, and 17, consistent with previous reports.^42^ Likewise, for CAMA-1 and MCF7, copy-number aberrations detected within cfChromatin were largely concordant with those from nuclear WGS (Figures S2B and S2C).^43^ Furthermore, these copy-number profiles were consistent across MNase digestion times (Figures S2E and S2F). Altogether, these results indicate that cfMNase-seq data reflect cancer-specific copy-number profiles and can be analyzed with existing plasma WGS tools.

### Simulated cfChromatin sequencing profiles at transcription start sites reflect transcriptional activity

Previous plasma WGS studies have demonstrated that cfChromatin sequencing coverage can be used to deduce nucleosome occupancy maps that, in turn, correlate with cellular transcription profiles.^14^^,^^19^^,^^23^^,^^24^ Specifically, at nucleosome-depleted regions (NDRs) of the genome, which tend to permit binding of transcriptional machinery, there is a reduction in sequencing coverage due to the digestion of unprotected DNA by plasma nucleases. Since nucleosome-protected fragments are over-represented in both plasma WGS and cfMNase-seq, we asked whether cfMNase-seq coverage also reflects associations with gene expression.

First, we obtained RNA sequencing (RNA-seq) data from all models and analyzed matched cfMNase-seq coverage surrounding transcription start sites (TSSs) (Figure 2B; Figures S3A–S3D). As anticipated, genes with high expression (e.g., >10 FPKM) displayed a prominent decrease in coverage at the TSS and large oscillations in coverage of flanking regions. This is consistent with strong positioning of nucleosomes upstream and downstream of NDRs.^19^^,^^23^^,^^24^ In contrast, genes with low expression did not show a pronounced decrease in coverage at the TSS and had weaker oscillations in coverage of flanking regions. While these profiles varied slightly across models, the correlation between gene expression and nucleosome positioning, quantified as the average normalized coverage between −125 and 25 bp centered around the TSS, was evident across all models with 30-min digestions (Spearman correlation ρ = −0.85, *p* = 3.5 × 10^−16^) (Figure 2C). Moreover, the association between gene expression and TSS normalized central coverage was present across varying digestion times (Figures S3C–S3F), though the correlation was stronger for CAMA-1 time course than HCT116 (Spearman correlation CAMA-1: ρ = −0.98, *p* = 2.8 × 10^−21^; HCT116: ρ = −0.54, *p* = 0.0022).

To further validate these findings, we confirmed that in the vicinity of the TSS, the genes with depletion of cfMNase-seq coverage corresponded to those with high assay for transposase-accessible chromatin using sequencing (ATAC-seq) signal in the corresponding cells, indicating that MNase preferentially degrades accessible TSSs (Figure S4A). Overall, these results illustrate how simulated cfChromatin sequencing profiles from preclinical models can reflect transcriptional activity by revealing nuclease-resistant nucleosome-bound regions.

### Simulated cfChromatin sequencing profiles beyond TSSs reveal cell-specific nucleosome organization

Having demonstrated the ability of cfChromatin sequencing profiles to reflect nucleosome organization associated with transcriptional activity, we next examined whether these profiles could reveal cancer-specific chromatin accessibility features distal to TSSs. We explored cfMNase-seq profiles at genome-wide chromatin accessibility variants that could distinguish between cell types^44^ (Figure S4B). Using ATAC-seq data, we performed a differential chromatin accessibility analysis and identified regions with enriched accessibility in each model versus all others, with the exception of the organoid models, which were grouped together due to lack of replicates and high correlation between samples (Figures S4B–S4D). The chromatin variants were predominantly found within introns and distal intergenic regions as opposed to the TSS (Figure S4C). We then evaluated cfMNase-seq coverage at these chromatin variants. For example, in regions with increased accessibility in A549, we observed a notable decrease in A549 cfMNase-seq coverage centered around the chromatin variants with prominent oscillations upstream and downstream, representing an NDR and strongly positioned flanking nucleosomes (Figure 2D). In contrast, the average cfMNase-seq signal across all other models remained fairly consistent across the A549 chromatin variants (Figure 2D). The normalized central coverage for each model at its chromatin variants with enriched accessibility compared to the others was notably different (paired Wilcoxon signed-rank test *p* = 0.063) (Figure 2E; Figure S4E), except for MCF7, where despite strong oscillations, the normalized central coverage was similar across models. Among sites with enriched chromatin accessibility in CAMA-1, distinct cfMNase-seq coverage profiles between CAMA-1 and the other models were maintained across all digestion times (Figure S4F). However, at regions with enriched chromatin accessibility in HCT116, the HCT116 cfMNase-seq coverage profiles varied notably across digestion times (Figure S4G), with the expected NDR coverage decrease appearing only with longer digestion times. Taken together, these results indicate that cfMNase-seq reflects nucleosome organization, chromatin accessibility, and molecular phenotypes that can be used to distinguish cell types.

### Simulated cfChromatin reflects cancer-specific chromatin features and overcomes signal dilution in low-burden patient plasma

To evaluate how simulated cfChromatin compares to plasma cfChromatin, we first analyzed xenograft plasma samples, which allow near-pure cancer-derived cfChromatin analysis through bioinformatic subtraction of mouse reads.^16^^,^^17^ Since simulated cfChromatin is also entirely cancer derived, xenograft plasma cfChromatin serves as a close comparison for investigating the effects of MNase—a bacterial nuclease—relative to the diverse nucleases responsible for fragmentation *in vivo*. To this end, we sequenced plasma cfChromatin from CAMA-1 xenograft mice and excluded mouse reads bioinformatically to isolate near-pure cancer-derived cfChromatin, which we refer to as xenograft plasma WGS.

We first assessed chromosomal variation across models and found strong concordance between xenograft plasma WGS copy-number profiles and those obtained from cfMNase-seq and nuclear WGS (Figures S2B and S5A). When examining nucleosome positioning in the vicinity of the TSS across gene expression levels, xenograft plasma WGS and cfMNase-seq produced highly similar profiles, with no significant difference in normalized central coverage metrics (Wilcoxon rank-sum test *p* = 0.77; Figure S5B). Both models displayed strong correlations between normalized central coverage and gene expression levels (Spearman correlation: simulated cfChromatin ρ = −0.98, *p* = 2.8 × 10^−21^; xenograft plasma WGS ρ = −0.98, *p* = 2.3 × 10^−7^; Figure S5C). Moreover, when analyzing chromatin variants enriched in CAMA-1, xenograft plasma WGS showed a numerical decrease in normalized central coverage (0.92) compared to the other models (0.99), though this reduction was less pronounced than that observed in CAMA-1 cfMNase-seq (0.78) (Figure S5D). Altogether, these results highlight that simulated cfChromatin captures chromosomal variation and nucleosome positioning patterns comparable to xenograft plasma WGS.

We next wanted to assess fragmentation features, namely fragment length and end motifs, which are more likely to be affected by the activity of distinct nucleases. CAMA-1 cfMNase-seq samples exhibited a high frequency of fragments corresponding to nucleosome-protected DNA (∼147 bp) (Figure S5E), while xenograft plasma WGS had a higher frequency of chromatosome-length fragments (∼166 bp) (Figure S5E). Furthermore, when interrogating 5′ 4-mer end-motifs, xenograft plasma WGS showed higher frequencies of C-end motifs, including CC-end motifs, which are associated with DNAse1L3 activity.^38^ Conversely, cfMNase-seq samples displayed an increase in A-end motifs with longer MNase digestion times (Figure S5F). End motifs from xenograft plasma WGS correlated most strongly with undigested cfChromatin samples (Figure S5G). These findings indicate that while simulated cfChromatin reflects nucleosomic features, it does not faithfully replicate certain *in vivo* fragmentation patterns.

**Figure 3:**
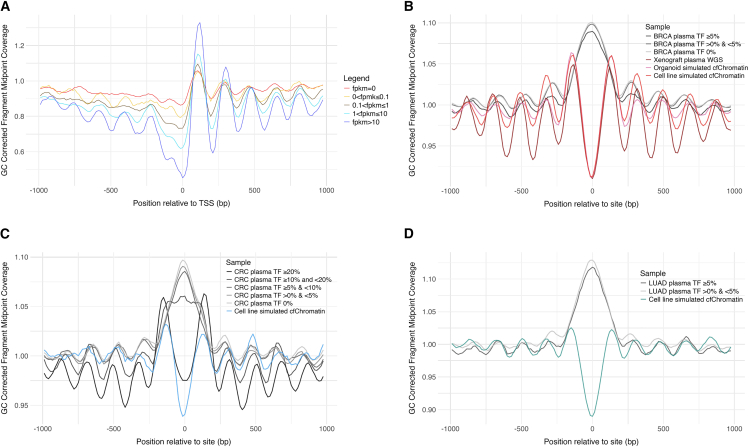
Simulated cfChromatin reflects cancer-specific chromatin features and overcomes signal dilution in low-burden patient plasma (A) Analysis of the association between CAMA-1 xenograft plasma WGS and gene expression around the TSS, shown for five binned FPKM levels. (B) Composite coverage profiles at 117,917 ATAC-seq open chromatin sites from independent breast cancer patient samples within TCGA after filtering out PBMC signal. Coverage profiles are shown for breast cancer patient plasma (*n* = 54) across varying tumor fractions (breakdown shown in Figure S6), simulated cfChromatin from organoids (BXTO.64, BPTO.95, and DCBPTO.66), simulated cfChromatin from cell lines (30-min digestion CAMA-1 and MCF7), and CAMA-1 xenograft plasma WGS. (C) Composite coverage profiles shown at 56,484 ATAC-seq open chromatin sites from independent colon adenocarcinoma patient samples within TCGA after filtering out PBMC signal. Composite profiles are shown for colorectal cancer patient plasma WGS (*n* = 27) and cell-line-simulated cfChromatin from HCT116 (30-min digestion). (D) Composite coverage profiles shown at 65,427 ATAC-seq open chromatin sites from independent lung adenocarcinoma patient samples within TCGA after filtering out PBMC signal. Composite profiles are shown for lung cancer patient plasma WGS (*n* = 9) and cell-line-simulated cfChromatin from A549. Composite site analysis for all individual samples and the corresponding metrics are in Figure S5. Within-figure abbreviations: BRCA, breast cancer; TF, tumor fraction; CRC, colorectal cancer; LUAD, lung adenocarcinoma.

Finally, we compared preclinical profiles to previously published plasma WGS data from preoperative treatment-naive breast (*n* = 54), colorectal (*n* = 27), and lung cancer (*n* = 9) patients.^45^ To reduce interference of cancer signals from hematopoietic-derived cfChromatin within patient samples, we filtered The Cancer Genome Atlas (TCGA) cancer ATAC-seq consensus peaks using peripheral blood mononuclear cell (PBMC) ATAC-seq dataset from 110 healthy volunteers.^46^ This approach enabled the identification of broad independent cancer-specific open chromatin regions distinct from hematopoietic-derived signals.^47^ Across these filtered regions, cfMNase-seq and xenograft plasma WGS—both near-pure cancer-derived cfChromatin sample types—showed decreased normalized central coverage and strong surrounding nucleosome positioning at open chromatin regions from the corresponding cancer type (Figures 3B–3D). In contrast, patient plasma WGS samples displayed an increase in coverage in these regions, reflecting their inaccessibility in most hematopoietic-derived cfChromatin after PBMC filtering (Figures 3B–3D; Figures S6A–S6G). Notably, colorectal cancer plasma samples with high tumor fractions (>20%) most closely mirrored the nucleosome profiles of preclinical samples, whereas samples with lower tumor fractions exhibited starkly different patterns (Figures 3B–3D; Figures S6A–S6G). We observed a weak negative correlation between tumor fraction and normalized central coverage across plasma samples (Spearman correlation: ρ = −0.2, *p* = 0.055), which would likely improve with additional high-burden samples and further refinement of chromatin accessibility regions of interest (Figure S6H). Altogether, these results highlight that simulated cfChromatin and xenograft plasma WGS sequencing profiles more closely reflect cancer-specific chromatin accessibility and nucleosome positioning than patient plasma WGS, where signal is diluted by non-cancer cfChromatin.

### H3K4me3 profiling from simulated cfChromatin reflects nuclear profiles and cell-specific biology

**Figure 4:**
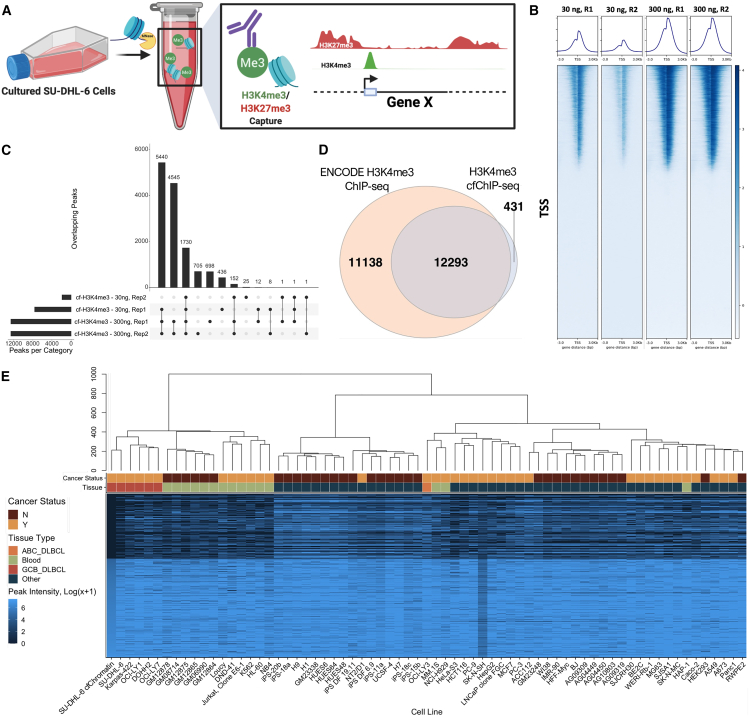
H3K4me3 profiling from simulated cfChromatin reflects cell line profiles and biology (A) Schematic summarizing cfChIP-seq methods for H3K4me3 and H3K27me3 from SU-DHL-6-simulated cfChromatin. (B) H3K4me3 cfChIP-seq coverage 3 kB around global TSSs, across biological replicates of 30 ng (low) and 300 ng (high) ChIP input concentrations. Profiles were subsampled down to the same read depth before analysis. (C) UpSet plot of the overlap in H3K4me3 cfChIP-seq MACS2-called peaks across replicate sets. The vertical bars represent the number of peaks shared among the sets specified by the connected circles below. Peak number for 30 ng, Rep2 was low due to low coverage. (D) Venn diagram representing the overlap in peaks for SU-DHL-6 between a H3K4me3 cfChIP-seq profile (300 ng, Rep1) and H3K4me3 nuclear ChIP-seq profile from the ENCODE database. (E) Heatmap of H3K4me3 peak signal intensity (log(x+1) transformed) from SU-DHL-6-simulated cfChromatin, SU-DHL-6 chromatin (ENCODE), other lymphomas (GCB and ABC DLBCLs), other blood-derived or adjacent cell types, and other non-blood cell types (e.g., pancreatic and prostate cancer and induced pluripotent stem cells). Cancer status indicates whether the sample is a cancer cell line (Y, yes, cancer cell line; N, no, non-cancer cell line). Each row represents the sum of peak intensities at a TSS (one TSS assigned per gene), and rows were sorted by SU-DHL-6 H3K4me3 cfChIP-seq peak intensity. Unsupervised hierarchical clustering was used to sort the samples based on the rows.

As nucleosome profiling indicated that simulated cfChromatin from tissue culture-conditioned media maintains the epigenetic state of the cell of origin, we utilized this framework to adapt and develop methods for the comprehensive profiling of genome-wide deposition of histone modifications associated with active and repressive chromatin states^48^ using cfChIP-seq (Figure 4A; Figure S7A). For this, we chose to study one of our previously analyzed models, SU-DHL-6, a germinal center B cell-like (GCB) diffuse large B cell lymphoma (DLBCL) cell line, because of the increasing relevance of epigenetic-targeted therapies in the treatment of lymphomas.^49^^,^^50^ First, we investigated H3K4me3, a histone modification that reflects active chromatin states at promoter regions responsible for transcriptional regulation,^51^ for which proof-of-concept studies have demonstrated the ability of cfChIP-seq to measure its genome-wide deposition within plasma.^25^ Within SU-DHL-6-simulated cfChromatin, H3K4me3 was sharply positioned around TSSs (Figure 4B). This signal was reproduced between replicates and across simulated cfChromatin input levels (Figure S7B). Peaks called by MACS2 were highly concordant (Figure 4C), with 97% of peaks identified by H3K4me3 cfChIP-seq also observed within SU-DHL-6 nuclear chromatin from ENCODE^52^ (Figure 4D).

Having validated the ability to profile H3K4me3 in simulated cfChromatin from our tissue culture system, we evaluated whether the H3K4me3 cfChIP-seq profiles were biologically representative of the cell line of interest, along with other similar cell types. We compared H3K4me3 cfChIP-seq peaks from SU-DHL-6 to H3K4me3 nuclear ChIP-seq peaks from a spectrum of cell types, including SU-DHL-6, other DLBCL cell lines (GCB and activated B cell [ABC] subtypes), other hematopoietic cell types, and a mix of differentiated and undifferentiated non-hematopoietic cell types (Figure 4E). Hierarchical clustering of peak intensities at TSSs revealed the similarity of GCB DLBCL profiles, while OCI-LY3, an ABC DLBCL, clustered separately. Cell types from other non-hematopoietic cell types clustered further away from the lymphoma profiles. We also obtained similar results when peaks were mapped to functional pathways prior to clustering (Figure S7C). Together, these results illustrate that H3K4me3 genome-wide deposition within simulated cfChromatin reflects cell-specific biological states.

### H3K27me3 from simulated cfChromatin reflects repressive chromatin states within SU-DHL-6 cells

**Figure 5:**
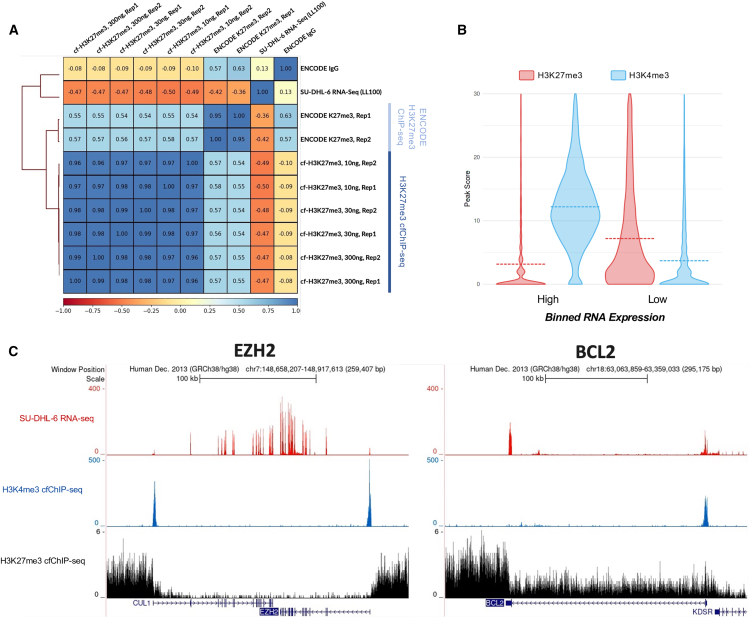
H3K27me3 profiling from simulated cfChromatin reflects repressive chromatin states within SU-DHL-6 cells (A) Genome-wide Spearman correlation (10 kb windows) between H3K27me3 cfChIP-seq profiles from SU-DHL-6-simulated cfChromatin (10, 30, and 300 ng input and technical replicates), ENCODE SU-DHL-6 H3K27me3 ChIP-seq profiles (biological replicates), SU-DHL-6 RNA-seq (LL100), and IgG control (ENCODE). (B) Violin plots depicting SU-DHL-6-simulated cfChromatin histone modification scores separated by median RNA-seq expression over 19,802 protein-coding TSSs. Violin plot median values are shown with dashed lines. *p* values (H3K4me3, ≤0.0001; H3K27me3, ≤0.0001) were calculated using a two-tailed *t* test. (C) Visualization of SU-DHL-6 RNA-seq, H3K4me3 cfChIP-seq, and H3K27me3 cfChIP-seq signal over EZH2 and BCL2, primary drivers of and prognostic factors in DLBCL, respectively.

While H3K4me3 cfChIP-seq represents a promising source of liquid biopsy biomarkers, we observed only modest variation across cell types, indicating that H3K4me3 alone may not entirely capture the complex epigenetic landscape of transcriptional regulation. To address this, we used simulated cfChromatin to develop and optimize methods for H3K27me3 cfChIP-seq. H3K27me3 is a heterochromatin mark that spans broad repressive domains associated with treatment response in a variety of cancers^53^^,^^54^^,^^55^ and may provide a new source of untapped liquid biopsy biomarkers. Genome-wide distributions of H3K27me3 within SU-DHL-6-simulated cfChromatin were strongly correlated between replicates and across simulated cfChromatin input levels (Spearman ρ = 0.96–0.99) and moderately correlated with SU-DHL-6 nuclear chromatin H3K27me3 but not IgG ChIP-seq from ENCODE^52^ (Figure 5A). A negative correlation was observed between H3K27me3 from simulated cfChromatin (Figure 5A, Spearman correlation, ρ = −0.47 to −0.50) and RNA-seq, which was stronger than the correlation observed for nuclear chromatin H3K27me3 (Figure 5A, Spearman correlation, ρ = −0.36 to −0.42), potentially due to reduced background signal from simulated cfChromatin (Figure S7D). Examining local gene features, H3K27me3 and RNA-seq^56^ profiles from SU-DHL-6 cells were negatively correlated, with high RNA expression accompanied by depletion of H3K27me3 from both simulated cfChromatin and nuclear chromatin at gene bodies, in contrast to the positive correlation with H3K4me3 observed at TSSs (Figures S7D–S7G).

The vast majority (94%) of peaks from SU-DHL-6 H3K27me3 cfChIP-seq profiles did not overlap with those from H3K4me3 cfChIP-seq (Figure S7H). Indeed, consistent with their known antagonistic roles in gene transcriptional regulation,^57^^,^^58^ these two marks had opposite associations with transcriptional activity in SU-DHL-6 (Figure 5B). Visualization of cfChIP-seq and RNA-seq profiles across *EZH2* and *BCL2*, representative genes with established functions in DLBCL,^59^^,^^60^ revealed depletion of H3K27me3 over the gene bodies and strong enrichment of H3K4me3 at the promoters (Figure 5C). Overall, these results demonstrate that simulated cfChromatin enables the development of robust methods for profiling repressive histone marks like H3K27me3. The resulting H3K27me3 profiles reflect expected distributions and characteristics of nuclear H3K27me3.

### Active and repressive histone modifications within simulated cfChromatin predict nuclear chromatin states and transcriptional programs

**Figure 6:**
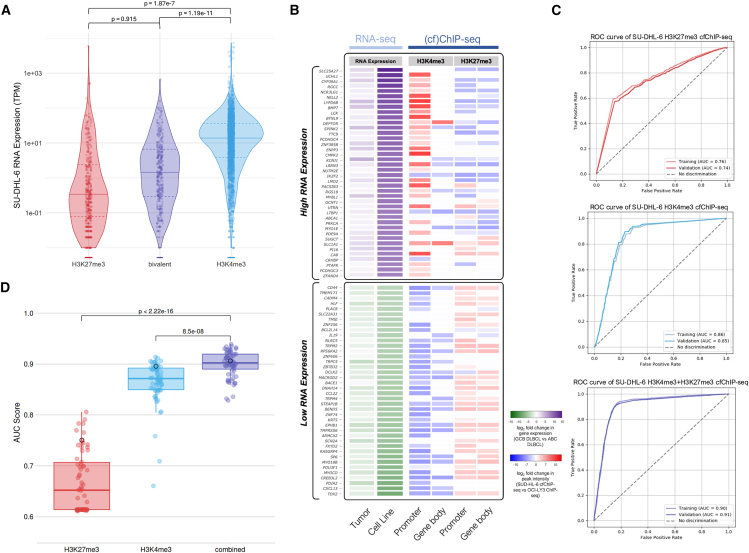
Active and repressive histone modifications within simulated cfChromatin predict nuclear chromatin states (A) Violin plots depicting SU-DHL-6 RNA-seq expression at genes marked with either promoter H3K27me3 only, H3K4me3 only, or both modifications (i.e., bivalent promoters). Peak groups were identified by quantifying the proportion of overlapping peaks from SU-DHL-6-simulated cfChromatin H3K4me3 and H3K27me3 profiles (Figure S4B). Only peaks within 3 kb of the TSS of the nearest gene were used in the analysis. Median RNA expression for each group is represented by a solid line, with the interquartile ranges marked by dotted lines. Welch’s *t* test: H3K4me3 vs. H3K27me3, *p* = 1.87e^−7^; H3K4me3 vs. bivalent, *p* = 1.19e^−11^; H3K27me3 vs. bivalent, *p* = 0.915. (B) Heatmap of gene expression and histone modification log2 fold change in 60 genes differentially expressed between GCB DLBCL and ABC DLBCL subtypes. Two independent differential gene expression analyses were performed, one comparing GCB (*n* = 34) and ABC DLBCL (*n* = 50) patient tumor samples and another comparing SU-DHL-6 (GCB subtype) and OCI-LY3 (ABC subtype). Pairwise average ranking between the tumor and cell line differentially expressed genes was performed to select the top 60 most significantly different based on *p* value. The log2 fold change in gene expression between GCB and ABC DLBCL subtypes is shown for patient tumor samples (first column) and the cell lines (second column). Differential analyses of histone modifications (H3K4me3 and H3K27me3) were then assessed across these genes by comparing SU-DHL-6 cfChIP-seq and OCI-LY3 ChIP-seq data. The log2 fold change in peak intensity between SU-DHL-6 and OCI-LY3 is shown for each histone modification in upstream promoter (third and fifth columns) and gene body (fourth and sixth columns) regions. (C) Receiver operating characteristic (ROC) curves for binary classification of high and low RNA gene expression levels using SU-DHL-6 cfChIP-seq. Logistic regression models trained and validated for H3K4me3, H3K27me3, and combined histone modifications. 10-fold cross-validation was performed using summed histone modification scores over the TSS and gene body of 19,802 protein-coding genes. Model performance was evaluated using the area under the curve (AUC). (D) Boxplots denoting AUC scores for binary classification of RNA gene expression levels across 56 cell types from the REMC database, 9 cell types from ENCODE, and SU-DHL-6-simulated cfChromatin (highlighted with a black outline). *p* value (combined-H3K4me3 = 8.5 × 10^−8^, combined-H3K27me3 < 2.2 × 10^−16^) was calculated using a paired *t* test.

In demonstrating that H3K27me3 in simulated cfChromatin reflects repressive states, we also observed a subset of regions bivalently marked by both H3K27me3 and H3K4me3 (Figure S7H). Among bivalently marked genomic locations within cfChromatin, the transcriptional activity of flanking genes varied widely with median levels between those of genes marked by only H3K27me3 or H3K4me3 (Figure 6A). An example of this is *TP53INP1* (Figure S7I), the expression of which has been shown to differentiate lymphomas with *EZH2* mutations and *BCL2* translocations from other lymphoma subtypes.^61^ Such bivalency—revealed only through integrated profiling of multiple histone modifications within cfChromatin—could represent a poised chromatin state compatible with timely adjustments upon developmental cues during tumorigenesis.^62^ These findings underscore the potential of simulated cfChromatin to enable the discovery of epigenetic biomarkers in cancer.

Building on these insights, we previously demonstrated that SU-DHL-6 (GCB DLBCL) and OCI-LY3 (ABC DLBCL) exhibited unique genome-wide deposition of H3K4me3 within simulated cfChromatin (Figure 4E). To further elucidate the molecular differences between these two subtypes, we analyzed differential gene expression in the cell line data and patient GCB and ABC DLBCL tumor samples^63^ (Figure 6B). Using genes with differential expression in both cell line and patient samples, we then assessed differential signal intensity from histone modification in the resulting regions. We observed that differentially upregulated genes in SU-DHL-6 relative to OCI-LY3 were enriched for H3K4me3 and diminished for H3K27me3 at their TSS and gene body. The opposite trends in histone modification patterns were observed for genes with lower expression in SU-DHL-6, demonstrating phenotypic characteristics that differentiate the cell lines reflected in simulated cfChromatin.

Having shown that the combination of active and repressive histone modifications within simulated cfChromatin may be complementary in reflecting chromatin state, we next assessed the ability of SU-DHL-6 H3K4me3 and H3K27me3 cfChIP-seq profiles to predict transcriptional activity. We adapted a binary classifier model from Frasca et al.^64^ to predict mRNA expression from histone modifications. SU-DHL-6 cfChromatin state profiles exhibited robust model performance for each modification and achieved a higher area under the receiver operating characteristic curve (AUC) when both H3K4me3 and H3K27me3 cfChIP-seq profiles were integrated into the model (Figure 6C). Next, to evaluate the performance of H3K27me3 and H3K4me3 to predict transcriptional activity across multiple cell types, we incorporated paired nuclear chromatin histone modification and RNA-seq data from Roadmap Epigenomics Mapping Consortium (56 cell types) and ENCODE (9 cell types). While AUC values were higher for H3K4me3 than for H3K27me3, the highest AUC values were achieved when incorporating both histone modifications (Figure 6D). Taken together, these results show the feasibility of predicting gene expression states using a minimal set of histone modifications, with simulated cfChromatin serving as a platform for developing these methods and uncovering biologically meaningful chromatin states that may be used to inform liquid biopsy biomarkers in patient samples.

## Discussion

This study focuses on the simulation and characterization of nucleosomal cfChromatin distributions from conditioned tissue culture media. Distinct from cfDNA, cfChromatin retains its association with the histone core complex and other chromatin-associated proteins, preserving the epigenetic modifications that govern chromatin states and associated DNA-templated processes, making it a profuse source of putative biomarkers. Motivated by limited sample availability and low abundance of tumor-derived cfChromatin in plasma, we sought to leverage cfChromatin in conditioned media. However, we observed a propensity of large cfChromatin fragments, which did not reflect the topology of plasma cfDNA, where mononucleosome-bound DNA is predominant, and which would not be compatible with many profiling methods. Accordingly, we developed an experimental framework using a nuclease treatment to generate nucleosome-sized cfChromatin consistently in both 2D and 3D culture systems, which we refer to as simulated cfChromatin. Simulated cfChromatin offers distinct advantages, including the absence of interfering signals from hematopoietic cells and other tissues, enabling the discovery of pure cancer-derived molecular features. Using this framework, we performed comprehensive genome-wide profiling of nucleosome positioning and active and repressive histone modifications. We demonstrate that as a renewable and reproducible source of cfChromatin, this system facilitates liquid biopsy method development and validation prior to testing with patient samples. Moreover, we compared simulated cfChromatin data types to nuclear chromatin profiles from the cell of origin, demonstrating that it faithfully reflects chromatin states and molecular features. Compared to nuclear chromatin, simulated cfChromatin exists in a fragmented, extracellular form suspended in liquid matrix, providing a practical and scalable model for liquid biopsy biomarker development. It is compatible with standard preprocessing workflows (e.g., cfDNA isolation) and analysis techniques (e.g., short read sequencing) used in liquid biopsy technologies, while also allowing repeated and easy sampling. Altogether, our study establishes simulated cfChromatin as a versatile tool for liquid biopsy method development and biomarker discovery, enabling further interrogation in patient samples.

In this work, we ascertained chromatin states within cfChromatin by conducting cfMNase-seq as well as cfChIP-seq for both H3K4me3 and H3K27me3. Through cfMNase-seq, we found that highly expressed genes displayed an NDR near their TSSs and strong positioning of surrounding nucleosomes, consistent with prior plasma WGS studies.^19^^,^^23^^,^^24^ Expanding beyond a gene-centric view, we identified genome-wide chromatin variants that generated distinct cfMNase-seq coverage profiles across most models. Notably, results were consistent across digestion conditions for CAMA-1, which contained a higher proportion of native mononucleosomes. In contrast, the HCT116 time course showed less pronounced NDR features at shorter digestion times, aligning with previous findings that higher levels of MNase digestion better reflect cfDNA nucleosome profiles.^17^ Future work can further explore digestion conditions relative to the abundance of native mononucleosomes across diverse models. Together, these results demonstrate that MNase-treated simulated cfChromatin captures key nucleosome positioning features, reinforcing its utility for studying cancer-derived cfChromatin in a liquid biopsy context.

To further validate our approach, we compared simulated cfChromatin with xenograft plasma WGS, a well-established source of near-pure cancer-derived cfChromatin for biomarker discovery (after bioinformatically removing reads mapped to the mouse genome),^16^^,^^34^ despite its resource-intensive nature and typical low yields of cancer-derived cfChromatin. We found that matched simulated cfChromatin and xenograft plasma WGS exhibited concordant chromosomal variation and nucleosome positioning patterns, reinforcing simulated cfChromatin as a comparable and more efficient alternative. In contrast, comparisons between our entirely cancer-derived cfChromatin samples and patient plasma WGS coverage at cancer-specific accessible chromatin sites (as determined by patient tumor tissue ATAC-seq data distinct from PBMCs) highlighted key differences. While cancer-specific regions of accessible chromatin in patient tumor samples corresponded with decreased cfMNase-seq and xenograft plasma WGS coverage, such pronounced coverage decreases were only observed in patient plasma WGS samples with high tumor fractions (>20%). This highlights the limitations of plasma WGS, where the low fractional abundance of tumor-derived cfChromatin is diluted by non-tumor sources. Conversely, the simulated cfChromatin analysis framework presented here provides unencumbered access to cancer-derived chromatin accessibility features without background contribution of signal from other cell types. Altogether, these results demonstrate that simulated cfChromatin reflects cancer-specific chromatin accessibility features while circumventing signal dilution observed in low-burden patient samples.

While using simulated cfChromatin to advance cfChIP-seq methods, this study explored cfChIP-seq profiles for histone modifications associated with both active and repressive chromatin states. As previously observed,^25^ the active mark H3K4me3 was sharply positioned at a subset of TSSs within simulated cfChromatin and was associated with high transcriptional activity. In contrast, the repressive mark H3K27me3 was more broadly distributed within SU-DHL-6-simulated cfChromatin and was associated with low transcriptional activity. Consequently, we observed a little overlap between the two marks: approximately 6% of H3K27me3 peaks and 9% of H3K4me3 peaks were shared in SU-DHL-6 cfChromatin. Promoters sharing these marks identified genes with a repressive RNA expression phenotype. These bivalent regions may be biologically significant, as they have been shown to be an integral part of cellular development and lineage specification.^59^^,^^65^

With growing evidence that subtyping lymphoma can improve patient outcomes,^66^ cfChIP-seq may offer new opportunities for clinical utility of epigenomic liquid biopsy biomarkers. Our results indicate that integrative profiling of both H3K4me3 and H3K27me3 may allow for more accurate cancer subtyping. The distribution of H3K4me3 within SU-DHL-6-simulated cfChromatin was most similar to nuclear chromatin ChIP-seq profiles from SU-DHL-6 and other GCB DLBCL cell lines, while being distinct from an ABC DLBCL cell line and non-hematopoietic cell types. Due to limited data availability from the ENCODE database, only a single ABC DLBCL profile was available for our study, and technical differences, such as antibody selection, may affect signal and limit direct comparability with simulated cfChromatin. Future studies can expand on this finding with paired cellular chromatin and simulated cfChromatin H3K4me3 profiles from an assortment of lymphoma cell lines to assess the capacity of these profiles to classify subtypes.

When H3K27me3 profiles were integrated into our analysis, we observed distinct histone modification patterns within genes that are differentially expressed between GCB and ABC DLBCL,^67^ including *LYPD6B*,^68^ *TOX2*,^69^ and *CD44*.^70^ Thus, we suggest that profiling histone modifications associated with both active and repressive chromatin states from cfChromatin could improve the phenotypic characterization of certain cancers that undergo substantial epigenomic dysregulation, resulting in significant changes in gene expression and chromosome stability.^71^ In the case of DLBCL, where epigenomic dysregulation is an important driver,^72^ integrative cfChIP-seq analysis may provide deeper biological insights and potential clinical utility.

In summary, we have developed a framework for the simulation of cfChromatin using conditioned media from cultured 2D and 3D models that better emulates cfChromatin topology in plasma. This framework provides readily accessible cfChromatin material, enabling liquid biopsy biomarker exploration without the contribution of background signal, which currently burdens exploratory analysis in human plasma samples. We also demonstrate the use of simulated cfChromatin in liquid biopsy profiling method development, as we adapted and developed multiple profiling approaches for evaluating various chromatin states, including cfMNase-seq and cfChIP-seq for H3K4me3 and H3K27me3. Altogether, our findings showcase the benefit of epigenetic profiling of cancer-derived cfChromatin from preclinical models. We intend for our framework of simulated cfChromatin and downstream profiling techniques to be applied in preclinical models to produce priors for biomarker discovery that can be tested in limited clinical samples. Areas for further exploration also include leveraging this framework to evaluate epigenetic changes associated with drug treatments, transformation, or differentiation over time with serial cfChromatin samples. Additionally, longer MNase digestion times and complementary approaches, such as single-stranded library preparation, may provide an opportunity to investigate cfChromatin topology. We anticipate that this work will accelerate the development of minimally invasive biomarkers applicable to cancer detection, classification, and treatment response prediction or monitoring, contributing to the future of precision medicine.

### Limitations of the study

Despite our efforts to validate the simulation of cfChromatin from tissue culture and its downstream epigenetic profiling, our work has certain limitations. First, this framework uses MNase, a bacteria-derived nuclease, which, as demonstrated with our comparison to xenograft plasma WGS, does not replicate all cfDNA fragmentation features generated by *in vivo* nucleases, such as DNase1L3 and DNase1.^38^ While we tested these alternative nucleases to produce nucleosome-sized cfChromatin distributions, they were suboptimal for this application. Future work may investigate the interplay of these nucleases in an attempt to better mimic *in vivo* fragmentation features. However, with MNase, the simulated cfChromatin framework should remain focused on interrogating nucleosome-related features, which were faithfully reproduced across a range of cancer models. Second, our findings align with previous literature indicating that variable experimental protocols, such as digestion time, may introduce biases that affect downstream nucleosome profiling.^17^^,^^20^^,^^73^ Standardized protocols for simulating cfChromatin should be followed to ensure reproducibility and consistent profiling of nucleosome features. Third, simulated cfChromatin from conditioned media does not capture the full physiological complexity of the plasma environment (e.g., circulatory dynamics), nor do we account for contributions from sources such as extracellular vesicles. Fourth, our study focused on a small number of preclinical models for most of the epigenomic profiling analyses. Future work should confirm the applicability of these techniques in models more representative of patient tumor biology and heterogeneity, including comparisons between patient-derived xenograft plasma and simulated cfChromatin from matched organoids or patient-derived cell lines. Fifth, we utilized low-coverage cfMNase-seq (median 2.5X), which inherently limited our ability to evaluate nucleosome footprints and gene expression at single-gene resolution that could be explored in deep sequencing datasets. Finally, we selected two histone modifications for cfChIP-seq profiling, whereas numerous others have been implicated in defining chromatin states relevant to cellular phenotypes^74^; future work could seek to incorporate other histone modifications, as well as DNA methylation^10^ and hydroxymethylation,^11^^,^^13^ into this simulated cfChromatin framework.

## Resource availability

### Lead contact

Any inquiries for additional information or resources should be addressed to the lead contact, Scott Bratman (scott.bratman@uhn.ca).

### Materials availability

This study did not generate new unique reagents.

### Data and code availability

• The cell line and xenograft plasma sequence data generated in this study will be publicly available at GEO (GSE242456) upon publication. The patient-derived organoid sequence data generated in this study are available under restricted access and will be released upon reasonable request, subject to a data transfer agreement. Visualizations of processed data files are available through the IGV web app at https://tinyurl.com/2xzlmtvy.
• This study also analyzed existing publicly available data. The MNase-seq FASTQ files were downloaded from the European Nucleotide Archive (https://www.ebi.ac.uk/ena/browser/view/SRX2349700 [SRR5022986 and SRR5022987]; https://www.ebi.ac.uk/ena/browser/view/SRX6066529 [SRR9297149]; https://www.ebi.ac.uk/ena/browser/view/SRX6066530 [SRR9297150]; and https://www.ebi.ac.uk/ena/browser/view/PRJNA310726 [SRR3142029–SRR3142036]).^39^^,^^40^ The WGS FASTQ files^43^ for CAMA-1 and MCF7 were downloaded (available at https://zenodo.org/records/6760819). The plasma WGS BAM files from treatment-naive breast, colorectal, and lung cancer patients were obtained from a previously published dataset^45^ and were downloaded from the European Genome-phenome Archive at the European Bioinformatics Institute (EGAD00001005339) with agreed use by the User Institution with a data access agreement. The tumor fractions for EGAD00001005339 were obtained from a previous publication^15^ that ran ichorCNA on the samples. The ATAC-seq FASTQ files for HCT116 (https://www.encodeproject.org/experiments/ENCSR872WGW/), MCF7 (https://www.encodeproject.org/experiments/ENCSR422SUG/), and A549 (https://www.encodeproject.org/experiments/ENCSR032RGS/) were downloaded from ENCODE.^52^ The ATAC-seq FASTQ files for SU-DHL-6 were downloaded from the European Nucleotide Archive (https://www.ebi.ac.uk/ena/browser/view/SRX12878040).^75^ The RNA-seq FASTQ files for HCT116 (https://www.encodeproject.org/experiments/ENCSR698RPL/) were downloaded from ENCODE. The RNA-seq FASTQ files for SU-DHL-6 ([SRR19938568],^76^ [ERR3003598]^56^) and OCILY3 ([SRR974972],^77^ [SRR17188140]^78^) were downloaded from Sequence Read Archive and European Nucleotide Archive. The RNA-seq data for BXTO.64 (BME ORG) and BPTO.95 (BME) were obtained from a previously published dataset.^79^ The RNA-seq data for 111 DLBCL specimens of formalin-fixed paraffin-embedded tissues were downloaded from NCBI (BioProject: PRJNA622950). The RNA-seq, H3K4me3, and H3K27me3 profiles from assorted cell types used in various analyses were downloaded from the Roadmap Epigenomics Consortium (Table S4) and ENCODE database (Table S5).
• The code used to generate figures in this manuscript will be available on Figshare (https://doi.org/10.6084/m9.figshare.24093627), following publication. Files required to run the analyses will also be available (https://doi.org/10.6084/m9.figshare.24093726). Any additional materials needed for the data analysis within this study can be provided by the lead contact upon request.

## Acknowledgments

We thank Jennifer Cruickshank for providing organoid conditioned media samples and Samah El Ghamrasni for sharing her insights with RNA-seq analysis. We thankfully acknowledge the Princess Margaret Genomics Center for carrying out next-generation sequencing and Zhibin Lu (University Health Network High-Performance Computing Center and Bioinformatics Core) for technical assistance. We also thank members of the Bratman laboratory for helpful discussions and suggestions. The graphical abstract and certain schematics were created using BioRender. S.V.B. is supported by the Gattuso-Slaight Personalized Cancer Medicine Fund at the Princess Margaret Cancer Centre and the Dr. Mariano Elia Chair in Head & Neck Cancer Research at the 10.13039/100009663University Health Network and the 10.13039/501100003579University of Toronto. S.D.D.M. was supported by a Canadian Institute of Health Research (CIHR) Fredrick Banting and Charles Best Canada Graduate Doctoral Scholarship (CGS-D, Application #457244). D.W.C. is supported by the 10.13039/501100000024Canadian Institutes of Health Research and the 10.13039/100009812Princess Margaret Cancer Foundation (DH Gales Family Foundation and the Green Fischer Family Trust and Goldie R. Feldman). S.C.M. and T.K. were previously supported by the Cancer Digital Intelligence Spark Award at the Princess Margaret Cancer Centre and the Ontario Graduate Scholarship at the 10.13039/501100003579University of Toronto. Currently, S.C.M. is supported by a Canadian Cancer Society Research Training Award – PhD (CCS award #708002). L.P. is supported by a Canadian Institute of Health Research (CIHR) Fredrick Banting and Charles Best Canada Graduate Doctoral Scholarship (CGS-D, Application #536349). M.L. is supported by the 10.13039/501100000024Canadian Institutes of Health Research (FRN-168933 and 191847), the Ontario Institute for Cancer Research (OICR) Investigator Award through funding provided by the 10.13039/100013873Government of Ontario (IA-031 to M.L.), and the 10.13039/100009812Princess Margaret Cancer Foundation and holds the Joey and Toby Tanenbaum/Brazilian Ball Chair. Research in the B.H.L. laboratory is funded by the 10.13039/501100004376Terry Fox Research Institute, Canada Foundation for Innovation, 10.13039/501100000024Canadian Institutes of Health Research, National Institutes of Health/10.13039/100000054National Cancer Institute (U01CA253383), and Clinical and Translational Science Center at Weill Cornell Medical Center, MSKCC (UL1TR00457).

## Author contributions

Conceptualization, S.D.D.M., S.C.M., and S.V.B.; methodology, S.D.D.M., S.C.M., A.A., and T.K.; formal analysis, S.D.D.M., S.C.M., L.P., and T.K.; investigation, S.D.D.M. and S.C.M.; resources, B.H.L., R.K., D.W.C., M.L., and S.V.B.; writing – original draft, S.D.D.M., S.C.M., and L.P.; writing – review and editing, S.D.D.M., S.C.M., L.P., A.A., T.K., B.H.L., R.K., D.W.C., M.M.H., M.L., and S.V.B.; visualization, S.D.D.M., S.C.M., and L.P.; supervision, S.V.B.; funding acquisition, S.V.B.

## Declaration of interests

S.V.B. is an inventor on patents related to cell-free DNA mutation and methylation analysis technologies that are unrelated to this work and have been licensed to Roche Molecular Diagnostics and Adela, respectively. S.V.B. is a co-founder of, has ownership in, and serves in a leadership role at Adela. M.M.H. is an inventor on a patent application related to cell-free methylation analysis licensed to Adela. D.W.C. reports consultancy and advisory relationships with AstraZeneca, Daiichi Sankyo, Exact Sciences, GenomeRx, Gilead, GlaxoSmithKline, Inivata/NeoGenomics, Lilly, Merck, Novartis, Pfizer, Roche, and SAGA and research funding to their institution from AstraZeneca, GenomeRx, Guardant Health, Grail, Gilead, GlaxoSmithKline, Inivata/NeoGenomics, Knight, Merck, PearBio, Pfizer, ProteinQure, and Roche. B.H.L. reports grants from Pfizer and grants, personal fees, and nonfinancial support from AstraZeneca, and personal fees from Daiichi Sankyo outside the submitted work. S.D.D.M. is a contractor working with Roche on minimally invasive cancer diagnostics.

## STAR★Methods

### Key resources table

**Table:** 

REAGENT or RESOURCE	SOURCE	IDENTIFIER
**Antibodies**		

Mouse monoclonal anti-Histone H3 (tri methyl K4)	Abcam	Cat# ab1012; RRID: AB_442796
Rabbit monoclonal anti-Histone H3, Trimethyl (Lys27)	Cell Signaling Technology	Cat# 9733S (lot 21); RRID: AB_2616029
IRDye 800CW goat polyclonal anti-Rabbit IgG (secondary)	LICORbio	Cat# 925-32211; RRID: AB_2651127
IRDye 800CW Goat polyclonal anti-Mouse IgG (secondary)	LICORbio	Cat# 925-32210; RRID: AB_2687825

**Biological samples**		

Patient derived organoids	Princess Margaret Cancer Center, University Health Network (UHN)	N/A
Human male genomic DNA	Promega	Cat# G1471

**Chemicals, peptides, and recombinant proteins**		

Recombinant Histone H3K4me1 (MLA)	Active Motif	Cat# 31208
Recombinant Histone H3K4me2 (MLA)	Active Motif	Cat# 31209
Recombinant Histone H3K4me3 (MLA)	Active Motif	Cat# 31600
Recombinant Histone H3K27me3 (MLA)	Active Motif	Cat# 31579
Matrigel	Corning	Cat# 356231
17β-estradiol 90-day slow-release pellet (0.72 mg)	Innovative Research of America	Cat# NE-121
DNase1L3	Aviva Systems Biology	Cat# OPCA03343
DNase1	New England Biolabs	Cat# M0303S
DNase1 buffer	New England Biolabs	Cat# B0303S
MNase & MNase buffer (included)	New England Biolabs	Cat# M0247S
BSA	New England Biolabs	Cat# B9000
EDTA (0.5 M, pH 8.0)	Invitrogen	Cat# 15575020
Low TE DNA suspension buffer (10 mM Tris-HCl, 0.1 mM EDTA, pH 8.0)	Teknova	Cat# T0220
AMPure XP Beads	Beckman Coulter	Cat# A63882
Dynabeads	Invitrogen	Cat# 14301

**Critical commercial assays**		

e-Myco™ VALiD Mycoplasma PCR Detection Kit	iNtRON Biotechnology	Cat# 25239
MinElute PCR Cleanup Kit	Qiagen	Cat# 28006
QIAamp Circulating Nucleic Acid Kit	Qiagen	Cat# 55114
Qubit High Sensitivity dsDNA Kit	Thermo Fisher Scientific (Life Technologies)	Cat# Q33231
Agilent High Sensitivity DNA Kit	Agilent Technologies	Cat# 5067-4626
SsoAdvanced Universal SYBR® Green Supermix	Bio-Rad	Cat# 1725274
KAPA HyperPrep Kit with Library Amplification Primer Mix	Roche	Cat# 07962363001

**Deposited data**		

cfMNase-seq, cfChIP-seq, ATAC-seq, RNA-seq, and xenograft plasma cfDNA WGS data	This paper	GSE242456
Additional data files used in this study	This paper	https://doi.org/10.6084/m9.figshare.24093726

**Experimental models: Cell lines**		

A549 (human lung adenocarcinoma)	Dr. Bradly Wouters	RRID:CVCL_0023
CAMA-1 (human breast adenocarcinoma)	Dr. David Cescon	RRID:CVCL_1115
HCT116 (human colorectal carcinoma)	Dr. Daniel De Carvalho	RRID:CVCL_0291
MCF7 (human breast adenocarcinoma)	Dr. David Cescon	RRID:CVCL_0031
SU-DHL-6 (human diffuse large B-cell lymphoma)	Dr. Robert Kridel	RRID:CVCL_2206

**Experimental models: Organisms/strains**		

Mouse: NOD/SCID IL2R-gamma-null (NSG)	The Jackson Laboratory	Cat# 005557; RRID:IMSR_JAX:005557

**Oligonucleotides**		

short human LINE-1 Forward Primer: 5′-TCACTCAAAGCCGCTCAACTAC-3′	IDT (Integrated DNA Technologies)	N/A
short human LINE-1 Reverse Primer: 5′-TCTGCCTTCATTTCGTTATGTACC-3′	IDT (Integrated DNA Technologies)	N/A
human GAPDH Forward Primer: 5′-GCCAATCTCAGTCCCTTCCC-3′	IDT (Integrated DNA Technologies)	N/A
human GAPDH Reverse Primer: 5′-TAGTAGCCGGGCCCTACTTT-3′	IDT (Integrated DNA Technologies)	N/A
human KAT6B Forward Primer: 5′-GAAGAGGCGGACCCAGCGGT-3′	IDT (Integrated DNA Technologies)	N/A
human KAT6B Reverse Primer: 5′-TTCCTGCCGGTCATCTCGCTT-3′	IDT (Integrated DNA Technologies)	N/A
human SLC22A3 Forward Primer: 5′-GGAGAGGGTGGACAGATTGA-3′	IDT (Integrated DNA Technologies)	N/A
human SLC22A3 Reverse Primer: 5′-TCAGCCTTGCTGCTACAGTG-3′	IDT (Integrated DNA Technologies)	N/A
human QML_93 Forward Primer: 5′-CACTGGTTGTCTTTGCAGGA-3′	IDT (Integrated DNA Technologies)	N/A
human QML_93 Reverse Primer: 5′-CCTGGGTCATATTGGGACAC-3′	IDT (Integrated DNA Technologies)	N/A

**Software and algorithms**		

Code used in this study	This paper	https://doi.org/10.6084/m9.figshare.24093627
FastQC (v0.11.9)	Babraham Bioinformatics	https://www.bioinformatics.babraham.ac.uk/projects/fastqc/; RRID:SCR_014583
UMI-tools (v1.1.1)	Smith et al.^80^	https://umi-tools.readthedocs.io/en/latest/; RRID:SCR_017048
Bowtie2 (v2.4.1)	Langmead and Salzberg^81^	https://bowtie-bio.sourceforge.net/bowtie2/index.shtml; RRID:SCR_016368
Samtools (v1.9)	Danecek et al.^82^	http://htslib.org/; RRID:SCR_002105
PDX_mouseSubtraction pipeline	Ha Lab; De Sarkar et al.^16^	https://github.com/GavinHaLab/PDX_mouseSubtraction
Cutadapt (v3.0)	Martin^83^	https://cutadapt.readthedocs.io/en/stable/; RRID:SCR_011841
Picard tools (v2.6.0)	Broad Institute	http://broadinstitute.github.io/picard/; RRID:SCR_006525
deepTools (v3.2.1)	Ramírez et al.^84^	https://deeptools.readthedocs.io/en/develop/; RRID:SCR_016366
MACS2 (v2.2.6, v2.2.7.1)	Zhang et al.^85^	https://github.com/macs3-project/MACS; RRID:SCR_013291
Chromatin accessibility pipeline	Lupien Lab	https://github.com/LupienLab/pipeline-chromatin-accessibility
Trim Galore! (v0.6.5, v0.6.6)	Babraham Bioinformatics	https://www.bioinformatics.babraham.ac.uk/projects/trim_galore/; RRID:SCR_011847
nf-core RNA-seq pipeline (v3.14.0)	Ewels et al.^86^	https://nf-co.re/rnaseq/3.14.0/; RRID:SCR_026973
STAR (v2.7.10a)	Dobin et al.^87^	https://github.com/alexdobin/STAR; RRID:SCR_004463
RSEM (v1.3.3)	Li and Dewey^88^	https://github.com/deweylab/RSEM; RRID:SCR_000262
TopHat2 (v2.1.1/2.0.x)	Kim et al.^89^	http://ccb.jhu.edu/software/tophat/index.shtml; RRID:SCR_013035
CyVerse Discovery Environment	Merchant et al.^90^	http://www.cyverse.org/; RRID:SCR_014531
UCSC Genome Browser	Kent et al.^91^	http://genome.ucsc.edu/; RRID:SCR_005780
R (v4.1.1, v4.2.1; including base packages such as stats)	R Core Team	http://www.r-project.org/; RRID:SCR_001905
ggplot2 (v3.4.0)	Wickham^92^	https://cran.r-project.org/web/packages/ggplot2/index.html; RRID:SCR_014601
Cluster (v2.1.4)	Maechler et al.	https://CRAN.R-project.org/package=cluster; RRID:SCR_013505
HMMcopy (readCounter)	Ha et al.^93^	https://bioconductor.org/packages/release/bioc/html/HMMcopy.html; RRID:SCR_026464
ichorCNA (v0.2.0)	Adalsteinsson et al.^41^	https://github.com/broadinstitute/ichorCNA; RRID:SCR_024768
DiffBind (v3.6.5, v3.8.4)	Stark and Brown^94^	http://bioconductor.org/packages/release/bioc/html/DiffBind.html; RRID:SCR_012918
edgeR	Ross-Innes et al.^95^	http://bioconductor.org/packages/edgeR/; RRID:SCR_012802
GenomicRanges (v1.54.1)	Lawrence et al.^96^	http://www.bioconductor.org/packages/2.13/bioc/html/GenomicRanges.html; RRID:SCR_000025
Griffin (v0.1.0)	Doebley et al.^15^	https://github.com/adoebley/Griffin
BioAnalyzer 2100 (v.B.02.10.SI764)	Agilent Technologies	https://www.agilent.com/en/product/automated-electrophoresis/bioanalyzer-systems/bioanalyzer-software/2100-expert-software-228259
ChIPPeakAnno (v3.28.0)	Bioconductor (Zhu et al.)	http://www.bioconductor.org/packages/release/bioc/html/ChIPpeakAnno.html; RRID:SCR_012828
UpSetR (v1.4.0)	CRAN (Conway et al.)	https://cran.r-project.org/web/packages/UpSetR/index.html; RRID:SCR_026112
HOMER (v4.6)	Heinz et al. 2010	http://homer.ucsd.edu/homer/; RRID: SCR_010881
dendextend (v1.16.0)	CRAN (Galili et al.)	https://cran.r-project.org/web/packages/dendextend/index.html; RRID:SCR_026116
bedtoolsr (v2.30.0-4)	Phanstiel Lab	https://github.com/PhanstielLab/bedtoolsr
BiomaRt (v2.50.0)	Bioconductor (Durinck et al.)	https://bioconductor.org/packages/biomaRt/; RRID:SCR_019214
ChIP-Enrich (v2.18.0)	Bioconductor (Welch et al.)	https://bioconductor.org/packages/release/bioc/html/chipenrich.html
DESeq2 (v1.36.0)	Love et al.^97^	https://bioconductor.org/packages/release/bioc/html/DESeq2.html; RRID:SCR_015687
ShallowChrome	Frasca et al.^64^	https://github.com/DEIB-GECO/ShallowChrome

**Other**		

K2 EDTA blood collection tubes	BD Biosciences	Cat# 365974
DNA LoBind 1.5 mL tubes	Eppendorf	Cat# 022-43-102-1
Mini-PROTEAN® TGX™ Precast Gel (4–20%)	Bio-Rad	Cat# 4568094

### Experimental model and study participant details

#### Mouse models

The animal experiments in this study were approved by the Animal Research Committee of the Princess Margaret Cancer Center (University Health Network) and performed in accordance with their regulations and guidelines. NOD/SCID IL2R-gamma-null (NSG, CAT #005557) female mice were purchased from Jackson Laboratory. All cell lines were subject to pathogen testing before experiments. Five million cells were injected in a 50% matrigel (Corning CAT #356231) solution into the mammary fat pad, and a 90-day slow-release estrogen pellet (0.72 mg of 17b-estradiol, Innovative Research of America, CAT #NE-121) was implanted between the ear and shoulder of the mouse to help ER + cancer cell line growth. At endpoint (maximum tumor size of 1.5 cm), blood was collected by terminal cardiac exsanguination under anesthesia (isoflurane inhalant) into K2 EDTA tubes (CAT #BD 365974). Blood was spun at 2,500*g* for 10 min at 4 °C, and plasma was transferred to 1.5 mL DNA LoBind Eppendorf tubes (CAT #022-43-102-1) and spun at 16,100*g* for 10 min at 4 °C. The double-spun plasma was transferred to a new 1.5 mL tube and stored at −80 °C until cfDNA isolation.

#### Culture models

All cell lines in this study were short tandem repeat tested to confirm the identity of the parent cell line. The CAMA-1 and MCF7 models used in this study were long-term passages. All cell lines were grown in their respective media (Table S1) supplemented with 10% fetal bovine serum (FBS) and 1% penicillin-streptomycin solution (Wisent Bioproducts). Organoids were cultured in a base medium of Dulbecco’s Modified Eagle Medium/Ham’s F-12 with exogenous growth factors, as previously described.^98^ All cell lines were mycoplasma tested before freezing down stocks and before experiments using the e-Myco VALiD Mycoplasma PCR Detection Kit (iNtRON Biotechnology, Inc., CAT #25239).

### Method details

#### Media sample collection and processing

Media samples were collected in the exponential growth phase, approximately three to four days after seeding. Media was placed in 15 mL conical tubes and spun at 2,500*g* for 10 min at 4 °C. Media was transferred to centrifuge-safe 5 mL Eppendorf tubes and spun at 16,100*g* for 10 min at 4 °C. The supernatant was transferred to a new 15 mL conical tube and kept at 4 °C for up to one week, prior to nucleosome preparation (without freezing).

#### Quantification of cfChromatin in culture media

To determine the concentration of cfChromatin in the media of each cell line, DNA was quantified using quantitative polymerase chain reaction (qPCR) against human LINE-1^99^^,^^100^ in quadruplicate. Media was diluted 1:100 in low TE DNA suspension buffer (10 mM Tris-HCl, 0.1 mM EDTA, pH 8.0, TEKnova, CAT #T0220). Human male genomic DNA (Promega, CAT #G1471) was used for a standard curve at 125 pg/μL, 12.5 pg/μL, 1.25 pg/μL, 0.125 pg/μL, and 0.0125 pg/μL. Each 10 μL reaction consisted of 4 μL template, 2x SsoAdvanced Universal SYBR Green Supermix (BioRad, CAT #1725274), and 2.5 μM oligonucleotides targeting hLINE-1 (Table S2).

#### Nuclease digestion of gDNA and cfChromatin

Purified, pre-sheared, and double size-selected naked genomic DNA (gDNA) from SW48 cells and media-derived cfChromatin from HCT116 cells were subjected to nuclease digestion under varying conditions using DNase1L3 (Aviva Systems Biology, CAT# OPCA03343), DNase1 (New England Biolabs, CAT# M0303S), and MNase (New England Biolabs, CAT# M0247S). For gDNA reactions, each nuclease was incubated with 50 ng of naked gDNA. DNase1L3 was tested at 12.5 μg/mL, 25 μg/mL, and 50 μg/mL in a reaction mix consisting of 10% DNase1 buffer (New England Biolabs, CAT# B0303S), 1% BSA (New England Biolabs, CAT# B9000), and molecular-grade water. DNase1 was tested at 20 U/mL, 100 U/mL, and 200 U/mL in a reaction mix containing 10% DNase1 buffer and molecular-grade water. MNase was tested at 100 U/mL, 500 U/mL, and 1000 U/mL in a reaction mix with 10% MNase buffer (New England Biolabs, CAT# M0247S), 1% BSA, and molecular-grade water. Reactions were incubated at 37°C for 30 min, and digestion was stopped by adding 5 mM EDTA (Invitrogen, CAT# 15575020). For DNase1 reactions, digestion was stopped with 10 min of incubation at 75°C. Negative controls were included for each condition, excluding the enzyme, to confirm DNA degradation was specific to nuclease activity. Purification of digested gDNA was performed using the Qiagen MinElute PCR Cleanup Kit (CAT# 28006). DNA yield was quantified using the Qubit High Sensitivity dsDNA Kit (Life Technologies, CAT# Q33231), and DNA fragment length distributions were analyzed using an Agilent 2100 Bioanalyzer with the High Sensitivity DNA Kit (CAT# 5067-4626), following the manufacturer’s instructions. For cfChromatin digestions, 200 ng of material was used. DNase1L3 was tested at final concentrations of 1.5 μg/mL, 3.125 μg/mL, 6.25 μg/mL, and 12.5 μg/mL. DNase1 was tested at 2 U/mL, 4 U/mL, 10 U/mL, and 20 U/mL, while MNase was tested at 133 U/mL, 500 U/mL, and 1000 U/mL. The reaction protocol and downstream analyses mirrored those for gDNA, with the exception of DNA isolation, which was performed using the Qiagen Circulating Nucleic Acid Kit (CAT# 55114).

#### Simulated cfChromatin generation with MNase

For preparing simulated cfChromatin from cell culture media, 1–5 mL conditioned media was subjected to digestion with a fixed concentration of MNase. Up to 400 ng/mL of cfChromatin input was tested using the fixed MNase condition. MNase master mix was prepared at 20,000 Units/mL (U/mL) by combining 440 μL of ddH20, 50 μL of MNase buffer, 5 μL of BSA, and 5 μL of 2,000,000 U/mL MNase (New England Biolabs, CAT #M0247S). Efforts were made to avoid freeze-thaw cycles of the MNase enzyme. MNase was added to cell line conditioned media supernatant at a final concentration of 133 U/mL and incubated in a 37 °C water bath for 30 min. The reaction was stopped by adding 5 mM EDTA (Invitrogen, CAT #15575020), and MNase-digested media was stored at −20 °C or processed immediately for cfDNA isolation or cfChIP-seq.

#### cfMNase-seq and xenograft plasma cfDNA sequencing

Purification of DNA from MNase-digested media cfChromatin and xenograft plasma cfChromatin was performed using the Qiagen Circulating Nucleic Acid kit (CAT #55114) and quantified using the Qubit High Sensitivity dsDNA kit (Life Technologies, CAT #Q33231) according to kit instructions. DNA fragment length distributions were analyzed on an Agilent 2100 Bioanalyzer with the High Sensitivity DNA kit (CAT #5067-4626) according to the manufacturer’s instructions. Purified cfDNA was stored at either 4°C (<3 days) or −20°C (>3 days). Illumina-compatible sequencing libraries were prepared from 20 ng of purified cfDNA in 50 μL of TE buffer for all models, except organoid and xenograft plasma samples were prepared from 10 ng and less than 10 ng, respectively. Libraries were prepared using the KAPA Hyperprep Kit with Library Amplification Primer Mix (Roche, CAT #07962363001) and six cycles of PCR amplification. Sequencing adapters containing unique molecular identifiers (UMIs) were obtained from Integrated DNA Technologies.^101^ Library clean-ups were performed using Beckman Coulter AMPure XP beads (CAT #A63882) with a 0.8X cleanup before library amplification and a 0.9X cleanup afterward. The library concentration was determined using the Qubit High Sensitivity dsDNA kit, and the fragment length distribution was assessed with an Agilent 2100 Bioanalyzer. Samples were pooled and sequenced (Table S3).

#### cfChIP-seq

Following generation of simulated cfChromatin, a predetermined quantity (10–300 ng SU-DHL-6 cfChromatin) was diluted in RPMI 1640 (Wisent Bioproducts, CAT #350-000-CL) and subjected to ChIP-seq adapted from Sadeh et al.^25^ Monoclonal antibodies targeting H3K4me3 (Abcam, ab1012) and H3K27me3 (Cell Signaling Technology, 9733S, lot 21) were first validated for antigen specificity by Western blot using recombinant H3K4me1, H3K4me2, H3K4me3, and H3K27me3. For the Western blots, 0.5 μg of recombinant histone was loaded per lane, and samples were run on a 4–20% polyacrylamide gradient gel (Bio-Rad, CAT #4568094) for 40 min at 100 V. Semi-dry transfer was utilized, and 1:1000 and 1:10000 dilutions of primary and secondary (LICORbio, CAT #925–32211/925-32210) antibodies, respectively, were used for detection.

To block any non-specific chromatin absorption during ChIP, Dynabeads (Invitrogen, CAT #14301) conjugated with antibodies were pre-incubated with 5% bovine serum albumin (BSA) in ddH2O for 1 h at room temperature. After one wash with media (without FBS) to remove residual BSA, the media sample and 3 μg dynabead-conjugated antibody were incubated overnight at 4°C. Beads were then washed prior to conducting on-bead library preparation as described^25^ using the KAPA Hyperprep Kit with Library Amplification Primer Mix. H3K4me3 libraries were amplified to 16 cycles, and H3K27me3 libraries were amplified to 12 cycles for 10–30 ng input and 11 cycles for 300 ng input. Library clean-ups were performed using AMPure XP beads with a 0.8X cleanup before library amplification and a 0.9X cleanup afterward. Library concentration was measured with the Qubit High Sensitivity dsDNA kit; fragment size distribution was evaluated by the Agilent 2100 Bioanalyzer. Samples were pooled and sequenced (Table S3).

#### ATAC-seq

Assay for transposase-accessible chromatin using sequencing (ATAC-seq) data was generated following a similar protocol as previously described.^102^ Briefly, frozen organoids were thawed in a cold douncer with 1 mL of homogenization buffer (HB) for 5 min. On ice, tissue was homogenized with pestle B for 10 strokes and filtered through a 40 μm tip into a chilled 1.5 mL Eppendorf tube. Nuclei were spun for 5 min at 500 g and 4°C in a fixed-angle centrifuge. The pellet with 50 μL of supernatant was kept and resuspended in 350 μL HB, then mixed with 400 μL of 50% Iodixanol solution. Next, 600 μL of 30% Iodixanol was layered on 600 μL of 40% Iodixanol, and 800 μL of the sample mixture was placed on top. The sample was spun in a swinging bucket centrifuge at 3000 g for 20 min, and 200 μL of nuclei were collected into a chilled 1.5 mL tube from the 30–40% interface. Nuclei were counted and 50,000 were used for the transposition reaction. For cancer cell lines, two biological replicates of 50,000 cells were lysed and used for the transposition reaction. An in-house Tn5 enzyme was used, prepared as previously described.^103^ Sample clean-up was performed with the Qiagen MinElute PCR Purification Kit (CAT #28004) with 250 μL of buffer PB for each 50 μL sample. A qPCR was then performed to determine the number of PCR amplification cycles to perform, and then the libraries were amplified (7–8 cycles). The samples were then cleaned up with the Qiagen MinElute PCR Purification Kit, diluted in 30 μL, and size selected with AMpure XP beads (0.5X). Lastly, a quality control qPCR was performed using 2.5 μL of diluted sample (6 μL sample in 30 μL water) and 7.5 μL of master mix (5 μL of 2X SYBR, 1 μL of 5 μM forward and reverse primers, and 2 μL of water) for two constitutively open (*GAPDH* and *KAT6B*) and closed regions (*QML93* and *SLC22A3*) (Table S2). All samples had fold enrichment greater than ten and were considered acceptable. The libraries were then quantified using a Qubit High Sensitivity dsDNA kit, and fragment length distribution was assessed with an Agilent 2100 Bioanalyzer. Libraries were pooled and underwent Illumina sequencing (Table S3).

#### RNA-seq

RNA was extracted from cells with NucleoSpin RNA kit (Machery Nagel). PolyA sequencing libraries were prepared and RNA-seq was conducted by Novogene Corporation (Table S3).

### Quantification and statistical analysis

#### Sequence data processing

##### cfMNase-seq

Paired-end cfMNase-seq reads were processed using a custom pipeline. Raw reads were quality-checked using FastQC (v0.11.9). UMIs were extracted using UMI-tools (v1.1.1) with a regular expression pattern to identify and append UMIs to the read headers.^80^ Processed reads were aligned to the hg38 human genome using Bowtie2 (v2.4.1).^81^ Duplicates were removed based on UMIs and alignment coordinates using UMI-tools. Low-quality mapped reads were filtered with Samtools view (v1.9) using a mapping quality threshold of 30.^82^ Quality metrics, including alignment statistics, were assessed at each step using Samtools flagstat.

##### Xenograft plasma WGS

Reads were processed with UMI-tools (v1.1.1) to extract UMIs, and then we followed a previously described protocol to remove mouse reads (https://github.com/GavinHaLab/PDX_mouseSubtraction, modified to use Bowtie2).^16^ Briefly, reads were aligned to a human (hg38) and mouse (mm10) concatenated genome using Bowtie2. Only read pairs where both reads aligned to the human reference genome were kept, and were realigned to a human-only reference prior to similar processing as the cfMNase-seq data.

##### cfChIP-seq

Paired-end sequencing reads were trimmed using Cutadapt (v3.0),^83^ utilizing a list of UMIs as input. Trimmed reads were aligned to the hg38 human genome build using Bowtie2 (v2.4.1).^81^ Duplicate reads were removed using Samtools (v1.9),^82^ using a combination of samtools sort (before and after samtools fixmate), samtools fixmate with the -m option, and samtools markdup with the -r option. Low-quality mapped reads were removed using samtools view with the -bq option, with a minimum mapping quality of 25–30. Fragment length distribution was assessed by Picard CollectInsertSizeMetrics (v2.6.0), and coverage was assessed with plotCoverage from deepTools (v3.2.1).^84^ Counts per 50 bp bin were calculated from the output BAM files (in bigWig format), using deepTools.^84^ Profiles that were not subsampled down for peak comparison were reads Per kilobase per million mapped reads (RPKM) normalized in bigWig format. Peaks were called using MACS2 (v2.2.7.1)^85^ using the narrow peak option for H3K4me3 and the broad peak option for H3K27me3. ENCODE blacklist regions were removed from peak sets and counts files.^104^

##### ATAC-seq

Reads were processed using a predefined pipeline (https://github.com/LupienLab/pipeline-chromatin-accessibility). Briefly, TrimGalore (v0.6.5) was used to trim FASTQ files, Bowtie2 (v2.3.5.1) was used for alignment, and MACS2 (v2.2.6) was used for peak calling. The analytical pipeline is also made reusable on the CoBE platform (www.pmcobe.ca/pipeline/60a4336aaf7a3251ac7e152d).

##### RNA-seq

RNA-sequencing datasets were processed using Nextflow (v24.10.1) nf-core RNA-seq pipelines (v3.14.0).^86^ Briefly, FASTQ files were aligned to hg38 using STARv2 aligner (v2.7.10a),^87^ and gene expression levels were quantified using RSEM (v1.3.3).^88^ Fragments per kilobase of transcript per million mapped reads (FPKM) and transcripts per million (TPM) normalized counts were used for downstream analyses. SU-DHL-6 RNA-seq^56^ was processed using TrimGalore (v0.6.6) for trimming (https://www.bioinformatics.babraham.ac.uk/projects/trim_galore/https://github.com/FelixKrueger/TrimGalore, implementable wrapper for CutAdapt^83^), TopHat2^89^ for alignment to GRCh38/hg38. Next, Samtools for removing duplicate reads, and deepTools to obtain RPKM normalized counts per 50 bp bin.^82^^,^^84^

#### Genome-wide cfMNase-seq and MNase-seq analysis

BigWig files with a 10,000 bp bin size were generated for cfMNase-seq samples in deepTools,^84^ uploaded to the CyVerse Discovery Environment,^90^ and visualized in the UCSC Genome Browser^91^ as a custom track. Next, the average scores for each bigWig file were computed across every genomic region using the multiBigWigSummary command in deepTools, producing a compressed NumPy array.^84^ A principal component analysis was performed on all cfMNase-seq and MNase-seq samples using the plotPCA command in deepTools,^84^ and was replotted in R (v4.2.1) using ggplot2 (v3.4.0).^92^ K-means clustering was performed using R-base packages, and a silhouette score was calculated per clustered group using Cluster (v2.1.4). The silhouette score is calculated as follows: for any point *i:*

$$s\left(i\right)=\frac{b\left(i\right)-a\left(i\right)}{\max\left(a\left(i\right),b\left(i\right)\right)}$$

Where *a(i)* represents the average intra-cluster distance between *i* and all other points in the cluster and *b(i)* represents the inter-cluster distance between *i* and the nearest cluster centroid; the average silhouette score is calculated for all data points within the dataset.

#### cfMNase-seq copy number aberration analysis

Copy number aberration analysis was conducted with HMMcopy readCounter (https://github.com/shahcompbio/hmmcopy_utils)^93^ and ichorCNA (v0.2.0).^41^ The analysis was run using the default parameters and default panel of healthy normal samples, except that we limited the analysis to autosomes, set estimateScPrevalence to false, and decreased the set of non-tumor fraction parameter restart values to (0.001,0.01,0.1,0.15,0.2,0.25) since our cfChromatin samples are entirely cancer-derived DNA. The default optimal solution was reported.

#### Reference file generation and coverage analysis

##### Gene expression reference files

Gene expression reference files were created using RNA-seq data for each respective cell line by subsetting transcripts by FPKM level (FPKM = 0, 0<FPKM≤0.1, 0.1<FPKM≤1, 1<FPKM≤10, FPKM>10).

##### Chromatin variant reference files

ATAC-seq BAM and narrowPeak files were used as input into DiffBind (v3.6.5)^94^ to identify sites of differential chromatin accessibility. Analysis followed methods previously described,^94^^,^^95^ and all parameters were set to defaults, except for normalization, which was set to background (https://bioconductor.org/packages/devel/bioc/vignettes/DiffBind/inst/doc/DiffBind.pdf). edgeR^105^ within DiffBind was used to select differential sites, and those with a false discovery rate less than 0.01 and a log2 fold change greater than two were considered differential. Reference site files were saved separately for differential open chromatin sites enriched in each model.

##### Cancer patient chromatin accessibility reference files

Open chromatin sites for primary breast cancer, colon adenocarcinoma, and lung adenocarcinoma patient samples from The Cancer Genome Atlas (TCGA) were downloaded from the TCGA ATAC-seq hub (https://gdc.cancer.gov/about-data/publications/ATACseq-AWG).^47^ A file with all cancer type-specific peak sets was downloaded (https://api.gdc.cancer.gov/data/71ccfc55-b428-4a04-bb5a-227f7f3bf91c), and files for breast cancer (BRCA_peakCalls.txt), colon cancer (COAD_peakCalls.txt), and lung cancer (LUAD_peakCalls.txt) were obtained. PBMC signal filtering was performed using GenomicRanges (v1.54.1)^96^ to exclude overlapping peaks from the PBMC consensus set^46^—generated by merging peaks across all PBMC samples—from the peak sets of each cancer type.

##### Coverage analysis

All reference files were formatted to be used as input site files for coverage evaluation with Griffin (v0.1.0).^15^ Griffin was run for cfMNase-seq samples and patient plasma WGS samples (*n* = 27 for colorectal cancer, *n* = 54 for breast cancer, *n* = 9 for lung cancer)^45^ with all default parameters, except for the normalized central coverage calculation region was set to −125 to −25 for TSSs evaluation, and the individual option was set as true. The coverage profiles were then plotted in R with ggplot2.^92^

#### cfMNase-seq and ATAC-seq comparison

A reference track file was downloaded from UCSC Table Browser^106^ with TSSs for all known genes. BigWig files were generated for ATAC-seq data and scores across TSSs were calculated using deepTools.^107^ The Griffin^15^ pipeline was then modified to extract GC-corrected bigWig files for cfMNase-seq coverage across the entire length of all fragments (as opposed to midpoint coverage for fragments 100–200 bp). The ATAC-seq signal sorted region files were then used to plot heatmaps in deepTools.

#### cfChIP-seq analysis

SU-DHL-6 simulated cfChromatin H3K4me3 profiles were visualized over TSSs using deepTools^107^ (v3.2.1), with the computeMatrix (reference-point, -a 3000, -b 3000 –skipZeros, and –missingDataAsZero options) and plotHeatmap functions. Correlation matrices were generated in deepTools using the multiBigwigSummary and plotCorrelation functions with either -pearson or -spearman options.

All statistic and analyses listed below using R statistical software were performed using R (v4.1.1). Stacked BioAnalyzer traces were re-plotted using ggplot2, using the raw output from the Agilent BioAnalyzer 2100 (v.B.02.10.SI764). Peak overlap analysis and Venn diagrams were done using ChIPPeakAnno (v3.28.0). UpSet plot was generated using UpSetR (v1.4.0), using the output from HOMER mergePeaks (https://github.com/stevekm/Bioinformatics/blob/776c420efac851c6780ce573939fb6610a3b9ae8/HOMER_mergePeaks_pipeline/HOMER_mergePeaks_venn_UpSetR/multi_peaks_UpSet_plot.R). Unsupervised hierarchical clustering was performed using the stats package (v4.1.1). Dendextend (v1.16.0) and bedtoolsr (v2.30.0-4) were used to sort samples and features in the custom heatmap for the H3K4me3 peak analysis. BiomaRt (v2.50.0) was used to generate the TSS reference for the custom heatmap. ChIP-Enrich (v2.18.0) was used to find the association between H3K4me3 peaks and active cellular pathways. Differential histone modification analysis between SU-DHL-6 cfChIP-seq and OCILY3 nuclear ChIP-seq samples was performed using DiffBind (v3.8.4)^95^ using default parameters as previously described.^94^^,^^95^

#### Differential mRNA Expression Analysis

Differential mRNA Expression Analysis was performed with DESeq2 (v1.36.0)^97^ using standard parameters in R (v4.2.1). Gene expression counts were computed as described above with GRCh38/hg38. Differential scores were calculated for germinal center B-cell-like against activated B-cell diffuse large B-cell lymphoma subtype and filtered for a minimum expression level of log_2_ ≥1.2 and FDR <0.05. This resulted in 314 upregulated and 1085 downregulated genes.

#### Supervised binary classification of SU-DHL-6 histone modifications

Binary classification of SU-DHL-6 gene expression from histone mark modifications was performed using an adapted version of ShallowChrome from Frasca et al.^64^ to predict gene expression. 56 cell types from Roadmap Epigenomics Consortium,^28^ 9 cell types from ENCODE,^52^^,^^108^^,^^109^^,^^110^,109–111 and a lab-generated cfChIP-seq SU-DHL-6 sample, each of which had paired histone modification and gene expression data, were used for this analysis and all were processed under ChIP-seq uniform processing guidelines as previously described^28^ (Table S4). This approach used peak calling within each gene’s TSS and gene body to predict gene expression for each given cell type. We applied 10-fold cross-validation of each cell type where approximately 6000 genes were held out in each fold and performance was computed using the area under the curve (AUC) for each individual gene feature and region combination.

## References

[1] Lone S.N., Nisar S., Masoodi T., Singh M., Rizwan A., Hashem S., El-Rifai W., Bedognetti D., Batra S.K., Haris M. (2022). Liquid biopsy: a step closer to transform diagnosis, prognosis and future of cancer treatments. Mol. Cancer.

[2] Lo Y.M.D., Han D.S.C., Jiang P., Chiu R.W.K. (2021). Epigenetics, fragmentomics, and topology of cell-free DNA in liquid biopsies. Science.

[3] Moss J., Magenheim J., Neiman D., Zemmour H., Loyfer N., Korach A., Samet Y., Maoz M., Druid H., Arner P. (2018). Comprehensive human cell-type methylation atlas reveals origins of circulating cell-free DNA in health and disease. Nat. Commun..

[4] Heitzer E., Haque I.S., Roberts C.E.S., Speicher M.R. (2019). Current and future perspectives of liquid biopsies in genomics-driven oncology. Nat. Rev. Genet..

[5] Ezeife D.A., Spackman E., Juergens R.A., Laskin J.J., Agulnik J.S., Hao D., Laurie S.A., Law J.H., Le L.W., Kiedrowski L.A. (2022). The economic value of liquid biopsy for genomic profiling in advanced non-small cell lung cancer. Ther. Adv. Med. Oncol..

[6] Newman A.M., Bratman S.V., To J., Wynne J.F., Eclov N.C.W., Modlin L.A., Liu C.L., Neal J.W., Wakelee H.A., Merritt R.E. (2014). An ultrasensitive method for quantitating circulating tumor DNA with broad patient coverage. Nat. Med..

[7] Zviran A., Schulman R.C., Shah M., Hill S.T.K., Deochand S., Khamnei C.C., Maloney D., Patel K., Liao W., Widman A.J. (2020). Genome-wide cell-free DNA mutational integration enables ultra-sensitive cancer monitoring. Nat. Med..

[8] Moser T., Kühberger S., Lazzeri I., Vlachos G., Heitzer E. (2023). Bridging biological cfDNA features and machine learning approaches. Trends Genet..

[9] Penny L., Main S.C., De Michino S.D., Bratman S.V. (2024). Chromatin and nucleosome-associated features in liquid biopsy: Implications for cancer biomarker discovery. Biochem. Cell. Biol..

[10] Shen S.Y., Singhania R., Fehringer G., Chakravarthy A., Roehrl M.H.A., Chadwick D., Zuzarte P.C., Borgida A., Wang T.T., Li T. (2018). Sensitive tumour detection and classification using plasma cell-free DNA methylomes. Nature.

[11] Füllgrabe J., Gosal W.S., Creed P., Liu S., Lumby C.K., Morley D.J., Ost T.W.B., Vilella A.J., Yu S., Bignell H. (2023). Simultaneous sequencing of genetic and epigenetic bases in DNA. Nat. Biotechnol..

[12] Baca S.C., Seo J.-H., Davidsohn M.P., Fortunato B., Semaan K., Sotudian S., Lakshminarayanan G., Diossy M., Qiu X., El Zarif T. (2023). Liquid biopsy epigenomic profiling for cancer subtyping. Nat. Med..

[13] Song C.X., Yin S., Ma L., Wheeler A., Chen Y., Zhang Y., Liu B., Xiong J., Zhang W., Hu J. (2017). 5-Hydroxymethylcytosine signatures in cell-free DNA provide information about tumor types and stages. Cell Res..

[14] Snyder M.W., Kircher M., Hill A.J., Daza R.M., Shendure J. (2016). Cell-free DNA Comprises an in Vivo Nucleosome Footprint that Informs Its Tissues-Of-Origin. Cell.

[15] Doebley A.-L., Ko M., Liao H., Cruikshank A.E., Santos K., Kikawa C., Hiatt J.B., Patton R.D., De Sarkar N., Collier K.A. (2022). A framework for clinical cancer subtyping from nucleosome profiling of cell-free DNA. Nat. Commun..

[16] De Sarkar N., Patton R.D., Doebley A.-L., Hanratty B., Adil M., Kreitzman A.J., Sarthy J.F., Ko M., Brahma S., Meers M.P. (2023). Nucleosome patterns in circulating tumor DNA reveal transcriptional regulation of advanced prostate cancer phenotypes. Cancer Discov..

[17] Rao S., Han A.L., Zukowski A., Kopin E., Sartorius C.A., Kabos P., Ramachandran S. (2022). Transcription factor–nucleosome dynamics from plasma cfDNA identifies ER-driven states in breast cancer. Sci. Adv..

[18] Yang X., Cai G.-X., Han B.-W., Guo Z.-W., Wu Y.-S., Lyu X., Huang L.-M., Zhang Y.-B., Li X., Ye G.-L., Yang X.X. (2021). Association between the nucleosome footprint of plasma DNA and neoadjuvant chemotherapy response for breast cancer. NPJ Breast Cancer.

[19] Ulz P., Thallinger G.G., Auer M., Graf R., Kashofer K., Jahn S.W., Abete L., Pristauz G., Petru E., Geigl J.B. (2016). Inferring expressed genes by whole-genome sequencing of plasma DNA. Nat. Genet..

[20] Shtumpf M., Piroeva K.V., Agrawal S.P., Jacob D.R., Teif V.B. (2022). NucPosDB: a database of nucleosome positioning in vivo and nucleosomics of cell-free DNA. Chromosoma.

[21] Jacob D.R., Guiblet W.M., Mamayusupova H., Shtumpf M., Ciuta I., Ruje L., Gretton S., Bikova M., Correa C., Dellow E. (2024). Nucleosome reorganisation in breast cancer tissues. Clin. Epigenet..

[22] Chereji R.V., Bryson T.D., Henikoff S. (2019). Quantitative MNase-seq accurately maps nucleosome occupancy levels. Genome Biol..

[23] Zhu G., Guo Y.A., Ho D., Poon P., Poh Z.W., Wong P.M., Gan A., Chang M.M., Kleftogiannis D., Lau Y.T. (2021). Tissue-specific cell-free DNA degradation quantifies circulating tumor DNA burden. Nat. Commun..

[24] Esfahani M.S., Hamilton E.G., Mehrmohamadi M., Nabet B.Y., Alig S.K., King D.A., Steen C.B., Macaulay C.W., Schultz A., Nesselbush M.C. (2022). Inferring gene expression from cell-free DNA fragmentation profiles. Nat. Biotechnol..

[25] Sadeh R., Sharkia I., Fialkoff G., Rahat A., Gutin J., Chappleboim A., Nitzan M., Fox-Fisher I., Neiman D., Meler G. (2021). ChIP-seq of plasma cell-free nucleosomes identifies gene expression programs of the cells of origin. Nat. Biotechnol..

[26] Fedyuk V., Erez N., Furth N., Beresh O., Andreishcheva E., Shinde A., Jones D., Zakai B.B., Mavor Y., Peretz T. (2023). Multiplexed, single-molecule, epigenetic analysis of plasma-isolated nucleosomes for cancer diagnostics. Nat. Biotechnol..

[27] Sipola J., Munzur A.D., Kwan E.M., Seo C.C.Y., Hauk B.J., Parekh K., Liao Y.J.R., Bernales C.Q., Donnellan G., Bloise I. (2025). Plasma cell-free DNA chromatin immunoprecipitation profiling depicts phenotypic and clinical heterogeneity in advanced prostate cancer. Cancer Res..

[28] Roadmap Epigenomics Consortium, Kundaje A., Meuleman W., Ernst J., Bilenky M., Yen A., Heravi-Moussavi A., Kheradpour P., Zhang Z., Wang J. (2015). Integrative analysis of 111 reference human epigenomes. Nature.

[29] Stetson D., Labrousse P., Russell H., Shera D., Abbosh C., Dougherty B., Barrett J.C., Hodgson D., Hadfield J. (2024). Next-Generation Molecular Residual Disease Assays: Do We Have the Tools to Evaluate Them Properly?. J. Clin. Oncol..

[30] Phallen J., Sausen M., Adleff V., Leal A., Hruban C., White J., Anagnostou V., Fiksel J., Cristiano S., Papp E. (2017). Direct detection of early-stage cancers using circulating tumor DNA. Sci. Transl. Med..

[31] Moding E.J., Nabet B.Y., Alizadeh A.A., Diehn M. (2021). Detecting Liquid Remnants of Solid Tumors: Circulating Tumor DNA Minimal Residual Disease. Cancer Discov..

[32] Bettegowda C., Sausen M., Leary R.J., Kinde I., Wang Y., Agrawal N., Bartlett B.R., Wang H., Luber B., Alani R.M. (2014). Detection of circulating tumor DNA in early- and late-stage human malignancies. Sci. Transl. Med..

[33] Abbosh C., Birkbak N.J., Swanton C. (2018). Early stage NSCLC — challenges to implementing ctDNA-based screening and MRD detection. Nat. Rev. Clin. Oncol..

[34] Rostami A., Lambie M., Yu C.W., Stambolic V., Waldron J.N., Bratman S.V. (2020). Senescence, Necrosis, and Apoptosis Govern Circulating Cell-free DNA Release Kinetics. Cell Rep..

[35] Ungerer V., Bronkhorst A.J., Van Den Ackerveken P., Herzog M., Holdenrieder S. (2021). Serial profiling of cell - free DNA and nucleosome histone modifications in cell cultures. Sci. Rep..

[36] Brind’Amour J., Liu S., Hudson M., Chen C., Karimi M.M., Lorincz M.C. (2015). An ultra-low-input native ChIP-seq protocol for genome-wide profiling of rare cell populations. Nat. Commun..

[37] Skene P.J., Henikoff S. (2017). An efficient targeted nuclease strategy for high-resolution mapping of DNA binding sites. eLife.

[38] Han D.S.C., Lo Y.M.D. (2021). The Nexus of cfDNA and Nuclease Biology. Trends Genet..

[39] Guzman C., D’Orso I. (2017). CIPHER: a flexible and extensive workflow platform for integrative next-generation sequencing data analysis and genomic regulatory element prediction. BMC Bioinf..

[40] Bacon C.W., Challa A., Hyder U., Shukla A., Borkar A.N., Bayo J., Liu J., Wu S.-Y., Chiang C.-M., Kutateladze T.G., D'Orso I. (2020). KAP1 Is a Chromatin Reader that Couples Steps of RNA Polymerase II Transcription to Sustain Oncogenic Programs. Mol. Cell.

[41] Adalsteinsson V.A., Ha G., Freeman S.S., Choudhury A.D., Stover D.G., Parsons H.A., Gydush G., Reed S.C., Rotem D., Rhoades J. (2017). Scalable whole-exome sequencing of cell-free DNA reveals high concordance with metastatic tumors. Nat. Commun..

[42] Passerini V., Ozeri-Galai E., de Pagter M.S., Donnelly N., Schmalbrock S., Kloosterman W.P., Kerem B., Storchová Z. (2016). The presence of extra chromosomes leads to genomic instability. Nat. Commun..

[43] Soria-Bretones I., Thu K.L., Silvester J., Cruickshank J., El Ghamrasni S., Ba-Alawi W., Fletcher G.C., Kiarash R., Elliott M.J., Chalmers J.J. (2022). The spindle assembly checkpoint is a therapeutic vulnerability of CDK4/6 inhibitor-resistant ER+ breast cancer with mitotic aberrations. Sci. Adv..

[44] Grillo G., Lupien M. (2022). Cancer-associated chromatin variants uncover the oncogenic role of transposable elements. Curr. Opin. Genet. Dev..

[45] Cristiano S., Leal A., Phallen J., Fiksel J., Adleff V., Bruhm D.C., Jensen S.Ø., Medina J.E., Hruban C., White J.R. (2019). Genome-wide cell-free DNA fragmentation in patients with cancer. Nature.

[46] Márquez E.J., Chung C.-H., Marches R., Rossi R.J., Nehar-Belaid D., Eroglu A., Mellert D.J., Kuchel G.A., Banchereau J., Ucar D. (2020). Sexual-dimorphism in human immune system aging. Nat. Commun..

[47] Corces M.R., Granja J.M., Shams S., Louie B.H., Seoane J.A., Zhou W., Silva T.C., Groeneveld C., Wong C.K., Cho S.W. (2018). The chromatin accessibility landscape of primary human cancers. Science.

[48] Zhao Z., Shilatifard A. (2019). Epigenetic modifications of histones in cancer. Genome Biol..

[49] Morschhauser F., Tilly H., Chaidos A., McKay P., Phillips T., Assouline S., Batlevi C.L., Campbell P., Ribrag V., Damaj G.L. (2020). Tazemetostat for patients with relapsed or refractory follicular lymphoma: an open-label, single-arm, multicentre, phase 2 trial. Lancet Oncol..

[50] He M.Y., Kridel R. (2021). Treatment resistance in diffuse large B-cell lymphoma. Leukemia.

[51] Wang H., Fan Z., Shliaha P.V., Miele M., Hendrickson R.C., Jiang X., Helin K. (2023). H3K4me3 regulates RNA polymerase II promoter-proximal pause-release. Nature.

[52] Consortium E.P., Dunham I., Kundaje A., Aldred S.F., Collins P.J., Davis C.a., Doyle F., Epstein C.B., Frietze S., Harrow J. (2013). An integrated encyclopedia of DNA elements in the human genome. Nature.

[53] Marsolier J., Prompsy P., Durand A., Lyne A.-M., Landragin C., Trouchet A., Bento S.T., Eisele A., Foulon S., Baudre L. (2022). H3K27me3 conditions chemotolerance in triple-negative breast cancer. Nat. Genet..

[54] Deblois G., Tonekaboni S.A.M., Grillo G., Martinez C., Kao Y.I., Tai F., Ettayebi I., Fortier A.-M., Savage P., Fedor A.N. (2020). Epigenetic switch-induced viral mimicry evasion in chemotherapy-resistant breast cancer. Cancer Discov..

[55] Gardner E.E., Lok B.H., Schneeberger V.E., Desmeules P., Miles L.A., Arnold P.K., Ni A., Khodos I., de Stanchina E., Nguyen T. (2017). Chemosensitive Relapse in Small Cell Lung Cancer Proceeds through an EZH2-SLFN11 Axis. Cancer Cell.

[56] Quentmeier H., Pommerenke C., Dirks W.G., Eberth S., Koeppel M., MacLeod R.A.F., Nagel S., Steube K., Uphoff C.C., Drexler H.G. (2019). The LL-100 panel: 100 cell lines for blood cancer studies. Sci. Rep..

[57] Barth T.K., Imhof A. (2010). Fast signals and slow marks: the dynamics of histone modifications. Trends Biochem. Sci..

[58] Yu J.-R., Lee C.-H., Oksuz O., Stafford J.M., Reinberg D. (2019). PRC2 is high maintenance. Genes Dev..

[59] Béguelin W., Popovic R., Teater M., Jiang Y., Bunting K.L., Rosen M., Shen H., Yang S.N., Wang L., Ezponda T. (2013). EZH2 is required for germinal center formation and somatic EZH2 mutations promote lymphoid transformation. Cancer Cell.

[60] Iqbal J., Neppalli V.T., Wright G., Dave B.J., Horsman D.E., Rosenwald A., Lynch J., Hans C.P., Weisenburger D.D., Greiner T.C. (2006). BCL2 expression is a prognostic marker for the activated B-cell--like type of diffuse large B-cell lymphoma. J. Clin. Oncol..

[61] Xu-Monette Z.Y., Wei L., Fang X., Au Q., Nunns H., Nagy M., Tzankov A., Zhu F., Visco C., Bhagat G. (2022). Genetic subtyping and phenotypic characterization of the immune microenvironment and MYC/BCL2 double expression reveal heterogeneity in diffuse large B-cell lymphoma. Clin. Cancer Res..

[62] Macrae T.A., Fothergill-Robinson J., Ramalho-Santos M. (2023). Regulation, functions and transmission of bivalent chromatin during mammalian development. Nat. Rev. Mol. Cell Biol..

[63] Yan W.-H., Jiang X.-N., Wang W.-G., Sun Y.-F., Wo Y.-X., Luo Z.-Z., Xu Q.-H., Zhou X.-Y., Cao J.-N., Hong X.-N., Li X.Q. (2020). Cell-of-Origin Subtyping of Diffuse Large B-Cell Lymphoma by Using a qPCR-based Gene Expression Assay on Formalin-Fixed Paraffin-Embedded Tissues. Front. Oncol..

[64] Frasca F., Matteucci M., Leone M., Morelli M.J., Masseroli M. (2022). Accurate and highly interpretable prediction of gene expression from histone modifications. BMC Bioinf..

[65] Madani Tonekaboni S.A., Haibe-Kains B., Lupien M. (2021). Large organized chromatin lysine domains help distinguish primitive from differentiated cell populations. Nat. Commun..

[66] Nowakowski G.S., Czuczman M.S. (2015). ABC, GCB, and Double-Hit Diffuse Large B-Cell Lymphoma: Does Subtype Make a Difference in Therapy Selection?. Am. Soc. Clin. Oncol. Educ. Book..

[67] Shipp M.A., Ross K.N., Tamayo P., Weng A.P., Kutok J.L., Aguiar R.C.T., Gaasenbeek M., Angelo M., Reich M., Pinkus G.S. (2002). Diffuse large B-cell lymphoma outcome prediction by gene-expression profiling and supervised machine learning. Nat. Med..

[68] Hodkinson B.P., Schaffer M., Brody J.D., Jurczak W., Carpio C., Ben-Yehuda D., Avivi I., Forslund A., Özcan M., Alvarez J. (2021). Biomarkers of response to ibrutinib plus nivolumab in relapsed diffuse large B-cell lymphoma, follicular lymphoma, or Richter’s transformation. Transl. Oncol..

[69] Zhou J., Toh S.H.-M., Tan T.K., Balan K., Lim J.Q., Tan T.Z., Xiong S., Jia Y., Ng S.-B., Peng Y. (2023). Super-enhancer-driven TOX2 mediates oncogenesis in Natural Killer/T Cell Lymphoma. Mol. Cancer.

[70] Tzankov A., Pehrs A.-C., Zimpfer A., Ascani S., Lugli A., Pileri S., Dirnhofer S. (2003). Prognostic significance of CD44 expression in diffuse large B cell lymphoma of activated and germinal centre B cell-like types: a tissue microarray analysis of 90 cases. J. Clin. Pathol..

[71] Baylin S.B., Jones P.A. (2016). Epigenetic Determinants of Cancer. Cold Spring Harb. Perspect. Biol..

[72] Jiang Y., Melnick A. (2015). The epigenetic basis of diffuse large B-cell lymphoma. Semin. Hematol..

[73] Chereji R.V., Ocampo J., Clark D.J. (2017). MNase-Sensitive Complexes in Yeast: Nucleosomes and Non-histone Barriers. Mol. Cell.

[74] Millán-Zambrano G., Burton A., Bannister A.J., Schneider R. (2022). Histone post-translational modifications - cause and consequence of genome function. Nat. Rev. Genet..

[75] Kodgule R., Goldman J.W., Monovich A.C., Saari T., Aguilar A.R., Hall C.N., Rajesh N., Gupta J., Chu S.-C.A., Ye L. (2023). ETV6 deficiency unlocks ERG-dependent microsatellite enhancers to drive aberrant gene activation in B-lymphoblastic leukemia. Blood Cancer Discov..

[76] Bal E., Kumar R., Hadigol M., Holmes A.B., Hilton L.K., Loh J.W., Dreval K., Wong J.C.H., Vlasevska S., Corinaldesi C. (2022). Super-enhancer hypermutation alters oncogene expression in B cell lymphoma. Nature.

[77] Hardee J., Ouyang Z., Zhang Y., Kundaje A., Lacroute P., Snyder M. (2013). STAT3 targets suggest mechanisms of aggressive tumorigenesis in diffuse large B-cell lymphoma. G3.

[78] Kwon D., Takata K., Zhang Z., Chong L., Fraser B., Zeisler J., Miyata-Takata T., Merkens H., Rousseau J., Aoki T. (2022). Targeting Refractory Mantle Cell Lymphoma for Imaging and Therapy Using C-X-C Chemokine Receptor Type 4 Radioligands. Clin. Cancer Res..

[79] Prince E., Cruickshank J., Ba-Alawi W., Hodgson K., Haight J., Tobin C., Wakeman A., Avoulov A., Topolskaia V., Elliott M.J. (2022). Biomimetic hydrogel supports initiation and growth of patient-derived breast tumor organoids. Nat. Commun..

[80] Smith T., Heger A., Sudbery I. (2017). UMI-tools: modeling sequencing errors in Unique Molecular Identifiers to improve quantification accuracy. Genome Res..

[81] Langmead B., Salzberg S.L. (2012). Fast gapped-read alignment with Bowtie 2. Nat. Methods.

[82] Danecek P., Bonfield J.K., Liddle J., Marshall J., Ohan V., Pollard M.O., Whitwham A., Keane T., McCarthy S.A., Davies R.M., Li H. (2021). Twelve years of SAMtools and BCFtools. GigaScience.

[83] Martin M. (2011). Cutadapt removes adapter sequences from high-throughput sequencing reads. EMBnet J..

[84] Ramírez F., Ryan D.P., Grüning B., Bhardwaj V., Kilpert F., Richter A.S., Heyne S., Dündar F., Manke T. (2016). deepTools2: a next generation web server for deep-sequencing data analysis. Nucleic Acids Res..

[85] Zhang Y., Liu T., Meyer C.A., Eeckhoute J., Johnson D.S., Bernstein B.E., Nusbaum C., Myers R.M., Brown M., Li W., Liu X.S. (2008). Model-based analysis of ChIP-Seq (MACS). Genome Biol..

[86] Ewels P.A., Peltzer A., Fillinger S., Patel H., Alneberg J., Wilm A., Garcia M.U., Di Tommaso P., Nahnsen S. (2020). The nf-core framework for community-curated bioinformatics pipelines. Nat. Biotechnol..

[87] Dobin A., Davis C.A., Schlesinger F., Drenkow J., Zaleski C., Jha S., Batut P., Chaisson M., Gingeras T.R. (2013). STAR: ultrafast universal RNA-seq aligner. Bioinformatics.

[88] Li B., Dewey C.N. (2011). RSEM: accurate transcript quantification from RNA-Seq data with or without a reference genome. BMC Bioinf..

[89] Kim D., Pertea G., Trapnell C., Pimentel H., Kelley R., Salzberg S.L. (2013). TopHat2: accurate alignment of transcriptomes in the presence of insertions, deletions and gene fusions. Genome Biol..

[90] Merchant N., Lyons E., Goff S., Vaughn M., Ware D., Micklos D., Antin P. (2016). The iPlant Collaborative: Cyberinfrastructure for Enabling Data to Discovery for the Life Sciences. PLoS Biol..

[91] Kent W.J., Sugnet C.W., Furey T.S., Roskin K.M., Pringle T.H., Zahler A.M., Haussler D. (2002). The human genome browser at UCSC. Genome Res..

[92] Hadley Wickham ggplot2: Elegant Graphics for Data Analysis. https://ggplot2.tidyverse.org./.

[93] Ha G., Roth A., Lai D., Bashashati A., Ding J., Goya R., Giuliany R., Rosner J., Oloumi A., Shumansky K. (2012). Integrative analysis of genome-wide loss of heterozygosity and monoallelic expression at nucleotide resolution reveals disrupted pathways in triple-negative breast cancer. Genome Res..

[94] Stark, R., and Brown, G. DiffBind : Differential binding analysis of ChIP- Seq peak data. http://bioconductor.org/packages/release/bioc/vignettes/DiffBind/inst/doc/DiffBind.pdf.

[95] Ross-Innes C.S., Stark R., Teschendorff A.E., Holmes K.A., Ali H.R., Dunning M.J., Brown G.D., Gojis O., Ellis I.O., Green A.R. (2012). Differential oestrogen receptor binding is associated with clinical outcome in breast cancer. Nature.

[96] Lawrence M., Huber W., Pagès H., Aboyoun P., Carlson M., Gentleman R., Morgan M.T., Carey V.J. (2013). Software for computing and annotating genomic ranges. PLoS Comput. Biol..

[97] Love M.I., Huber W., Anders S. (2014). Moderated estimation of fold change and dispersion for RNA-seq data with DESeq2. Genome Biol..

[98] Sachs N., de Ligt J., Kopper O., Gogola E., Bounova G., Weeber F., Balgobind A.V., Wind K., Gracanin A., Begthel H. (2018). A Living Biobank of Breast Cancer Organoids Captures Disease Heterogeneity. Cell.

[99] Rago C., Huso D.L., Diehl F., Karim B., Liu G., Papadopoulos N., Samuels Y., Velculescu V.E., Vogelstein B., Kinzler K.W., Diaz L.A. (2007). Serial Assessment of Human Tumor Burdens in Mice by the Analysis of Circulating DNA. Cancer Res..

[100] Rostami A., Yu C., Bratman S.V. (2020). Serial Cell-free DNA Assessments in Preclinical Models. STAR Protoc..

[101] Wang T.T., Abelson S., Zou J., Li T., Zhao Z., Dick J.E., Shlush L.I., Pugh T.J., Bratman S.V. (2019). High efficiency error suppression for accurate detection of low-frequency variants. Nucleic Acids Res..

[102] Corces M.R., Trevino A.E., Hamilton E.G., Greenside P.G., Sinnott-Armstrong N.A., Vesuna S., Satpathy A.T., Rubin A.J., Montine K.S., Wu B. (2017). An improved ATAC-seq protocol reduces background and enables interrogation of frozen tissues. Nat. Methods.

[103] Picelli S., Björklund A.K., Reinius B., Sagasser S., Winberg G., Sandberg R. (2014). Tn5 transposase and tagmentation procedures for massively scaled sequencing projects. Genome Res..

[104] Amemiya H.M., Kundaje A., Boyle A.P. (2019). The ENCODE Blacklist: Identification of Problematic Regions of the Genome. Sci. Rep..

[105] Robinson M.D., McCarthy D.J., Smyth G.K. (2010). edgeR: a Bioconductor package for differential expression analysis of digital gene expression data. Bioinformatics.

[106] Karolchik D., Hinrichs A.S., Furey T.S., Roskin K.M., Sugnet C.W., Haussler D., Kent W.J. (2004). The UCSC Table Browser data retrieval tool. Nucleic Acids Res..

[107] Ramírez F., Ryan D.P., Grüning B., Bhardwaj V., Kilpert F., Richter A.S., Heyne S., Dündar F., Manke T. (2016). deepTools2: a next generation web server for deep-sequencing data analysis. Nucleic Acids Res..

[108] Luo Y., Hitz B.C., Gabdank I., Hilton J.A., Kagda M.S., Lam B., Myers Z., Sud P., Jou J., Lin K. (2020). New developments on the Encyclopedia of DNA Elements (ENCODE) data portal. Nucleic Acids Res..

[109] Kagda M.S., Lam B., Litton C., Small C., Sloan C.A., Spragins E., Tanaka F., Whaling I., Gabdank I., Youngworth I. (2023). Data navigation on the ENCODE portal. arXiv.

[110] Hitz B.C., Lee J.-W., Jolanki O., Kagda M.S., Graham K., Sud P., Gabdank I., Seth Strattan J., Sloan C.A., Dreszer T. (2023). The ENCODE Uniform Analysis Pipelines. bioRxiv.

