# Supplementary material for: Simulating cell-free chromatin using preclinical cancer models for liquid biopsy applications
Source: iScience. 2025 Nov 22;28(12):114113. doi: 10.1016/j.isci.2025.114113 (PMC12767190; doi:10.1016/j.isci.2025.114113)
Supplement: Document S1. Figures S1–S7 and Tables S1–S5 [file mmc1.pdf]

iScience, Volume *28*

Supplemental information

Simulating cell-free chromatin using preclinical cancer models for liquid biopsy applications

Sasha C. Main, Steven D. De Michino, Lucas Penny, Aleem Aamir, Tina Keshavarzian, Benjamin H. Lok, Robert Kridel, David W. Cescon, Michael M. Hoffman, Mathieu Lupien, and Scott V. Bratman

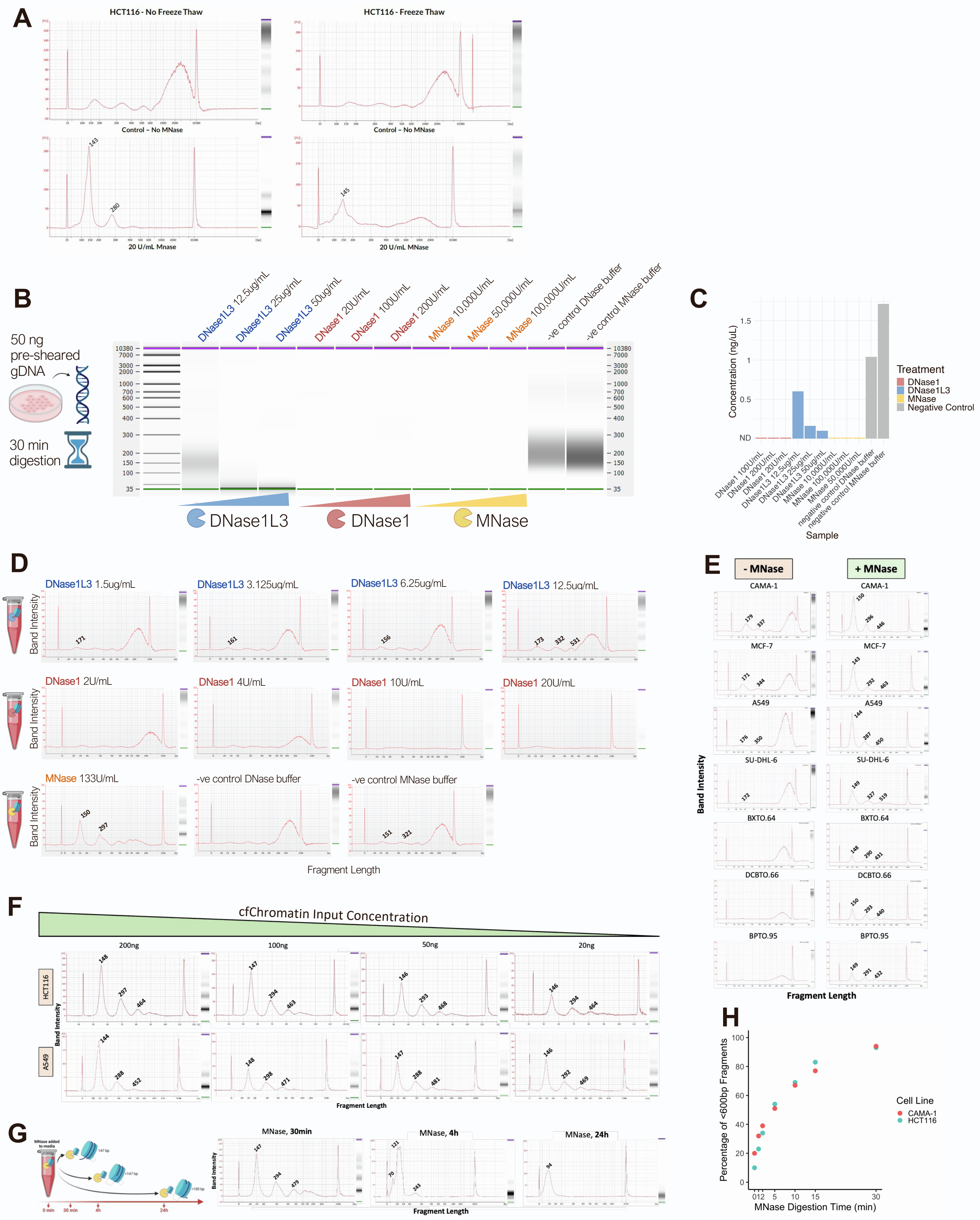
A
HCT116 - No Freeze Thaw
HCT116 - Freeze Thaw
Control – No MNase
20 U/mL Mnase
20 U/mL MNase
B
50 ng pre-sheared gDNA
30 min digestion
DNase1L3 12.5ug/mL
DNase1L3 25ug/mL
DNase1L3 50ug/mL
DNase1 20U/mL
DNase1 100U/mL
DNase1 200U/mL
MNase 10,000U/mL
MNase 50,000U/mL
MNase 100,000U/mL
-ve control DNase buffer
-ve control MNase buffer
DNase1L3
DNase1
MNase
C
Concentration (ng/uL)
Treatment
DNase1
DNase1L3
MNase
Negative Control
Sample
D
DNase1L3 1.5ug/mL
DNase1L3 3.125ug/mL
DNase1L3 6.25ug/mL
DNase1L3 12.5ug/mL
DNase1 2U/mL
DNase1 4U/mL
DNase1 10U/mL
DNase1 20U/mL
MNase 133U/mL
-ve control DNase buffer
-ve control MNase buffer
Band Intensity
Fragment Length
E
- MNase
+ MNase
CAMA-1
MCF-7
A549
SU-DHL-6
BXTO.64
DCBTO.66
BPTO.95
Band Intensity
Fragment Length
F
cfChromatin Input Concentration
200ng
100ng
50ng
20ng
HCT116
A549
Band Intensity
Fragment Length
G
MNase added to media
0 min
30 min
4h
24h
MNase, 30min
MNase, 4h
MNase, 24h
Band Intensity
Fragment Length
H
Percentage of <600bp Fragments
MNase Digestion Time (min)
Cell Line
CAMA-1
HCT116

Supplementary Figure 1, related to Figure 1. Methodological considerations for MNase treatment of media cfChromatin

(A) Results of Agilent BioAnalyzer analysis are shown. HCT116 culture media cfChromatin was treated with MNase before and after media freeze-thaw compared to untreated conditions (left and right, respectively). (B) 50 ng of SW48 pre-sheared genomic DNA (gDNA) was digested with varying concentrations of DNase1L3 (12.5-50ug/mL), DNase1 (20-200U/mL), and MNase (10,000-100,000U/mL) for 30 minutes to confirm enzymatic activity. Results of Agilent BioAnalyzer gel are shown, depicting progressive digestion with DNase1L3 as the concentration increases, and complete DNA digestion for all concentrations of DNase1 and MNase, compared to two negative controls. (C) Concentrations determined by Qubit post SW48 gDNA nuclease digestion for DNase1L3, DNase1, MNase, and two negative controls. (D) 200 ng of HCT116 culture media cfChromatin was treated with DNase1L3 (1.5, 3.125, 6.25, 12.5 ug/mL), DNase1 (2, 4, 10, 20 U/mL), and MNase (133 U/mL). Results of Agilent BioAnalyzer analysis are shown, demonstrating minimal nucleosome production with DNase1L3 with tested concentrations, and lack of nucleosome-sized fragments produced with DNase1. MNase treatment of cfChromatin generated a high proportion of nucleosome-sized fragments compared to the negative controls. (E) Comparison of MNase vs no MNase conditions for all cell lines and breast cancer organoid models are shown. (F) Different concentrations of HCT116 media cfChromatin (ranging from 20-200 ng) were treated with MNase in 1 mL volumes, respectively. Bioanalyzer analysis after cfDNA purification was performed. (G) HCT116 media was treated with MNase and the reaction halted after 30 minutes, four hours, and 24 h. After 30 minutes, mono-, di- and tri-nucleosomes are present. As digestion continues, oligonucleosome fragments are degraded, and MNase continues to digest mono-nucleosomes within the nucleosome core. (H) Progressive nucleosome production over time is consistent across HCT116 and CAMA-1 media, with a higher rate of nucleosomes produced at earlier time points.

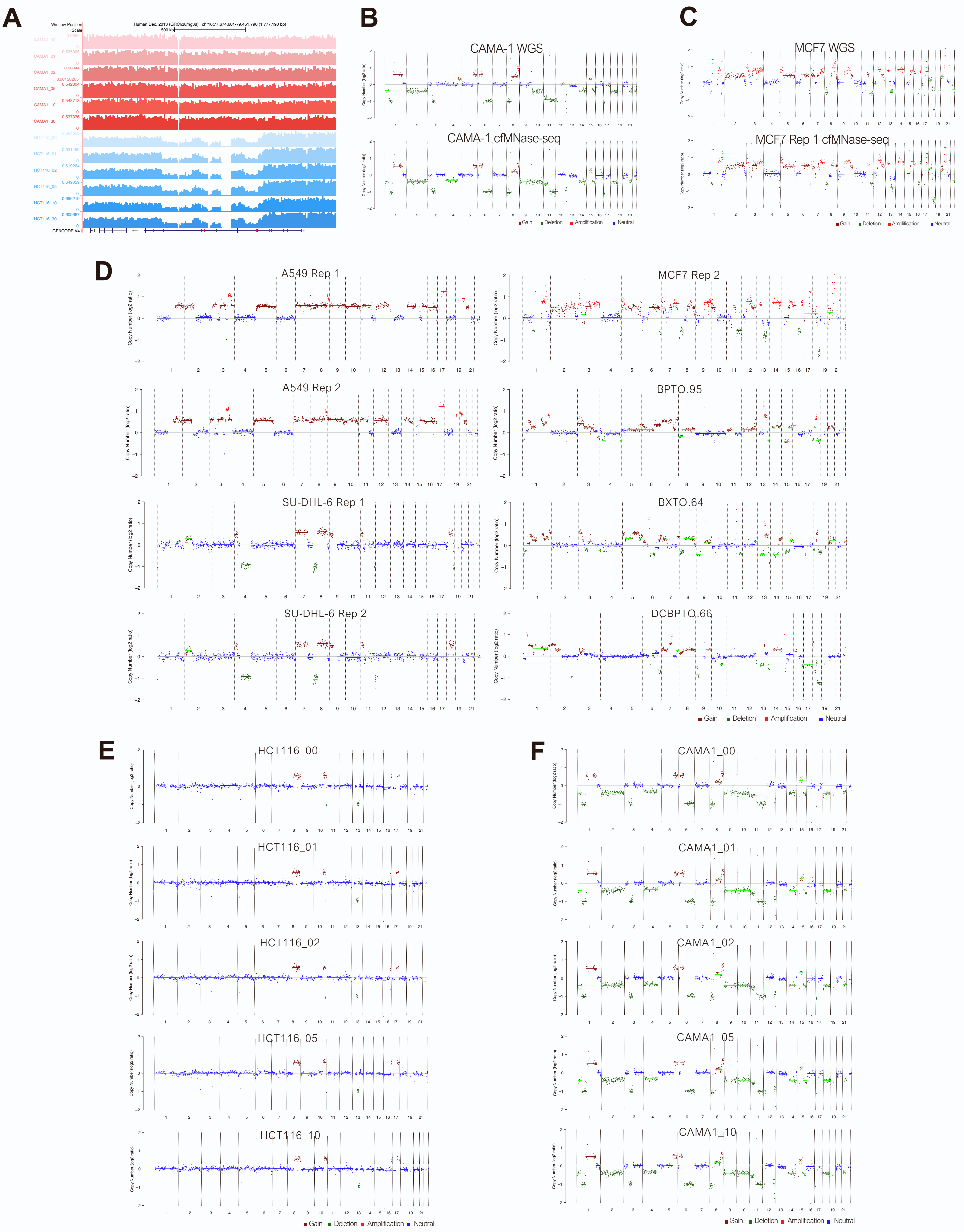
A
B
CAMA-1 WGS
CAMA-1 cfMNase-seq
C
MCF7 WGS
MCF7 Rep 1 cfMNase-seq
D
A549 Rep 1
MCF7 Rep 2
A549 Rep 2
BPTO.95
SU-DHL-6 Rep 1
BXTO.64
SU-DHL-6 Rep 2
DCBPTO.66
E
HCT116_00
HCT116_01
HCT116_02
HCT116_05
HCT116_10
F
CAMA1_00
CAMA1_01
CAMA1_02
CAMA1_05
CAMA1_10
Copy Number (log2 ratio)
Gain
Deletion
Amplification
Neutral

Supplementary Figure 2, related to Figure 2. Genome-wide cfMNase-seq profiles are consistent across different digestion times and concordant with nuclear signal

(A) A representation of the 10,000bp bin size resolution through a UCSC Genome Browser view across cfMNase-seq time course samples. This bin size was used for genome-wide comparisons across CAMA-1 and HCT116 cfMNase-seq time course samples. (B) Copy number analysis comparison between WGS data and cfMNase-seq (30-minute digestion) for CAMA-1 and (C) for MCF7. (D) Copy number analysis using ichorCNA with cfMNase-seq data across other models (A549, SU-DHL-6, MCF7, BPTO.95, BXTO.64, and DCBPTO.66) and (E) across various digestion times (no MNase to 30-minute digestion, from top to bottom) for HCT116 and (F) CAMA-1.

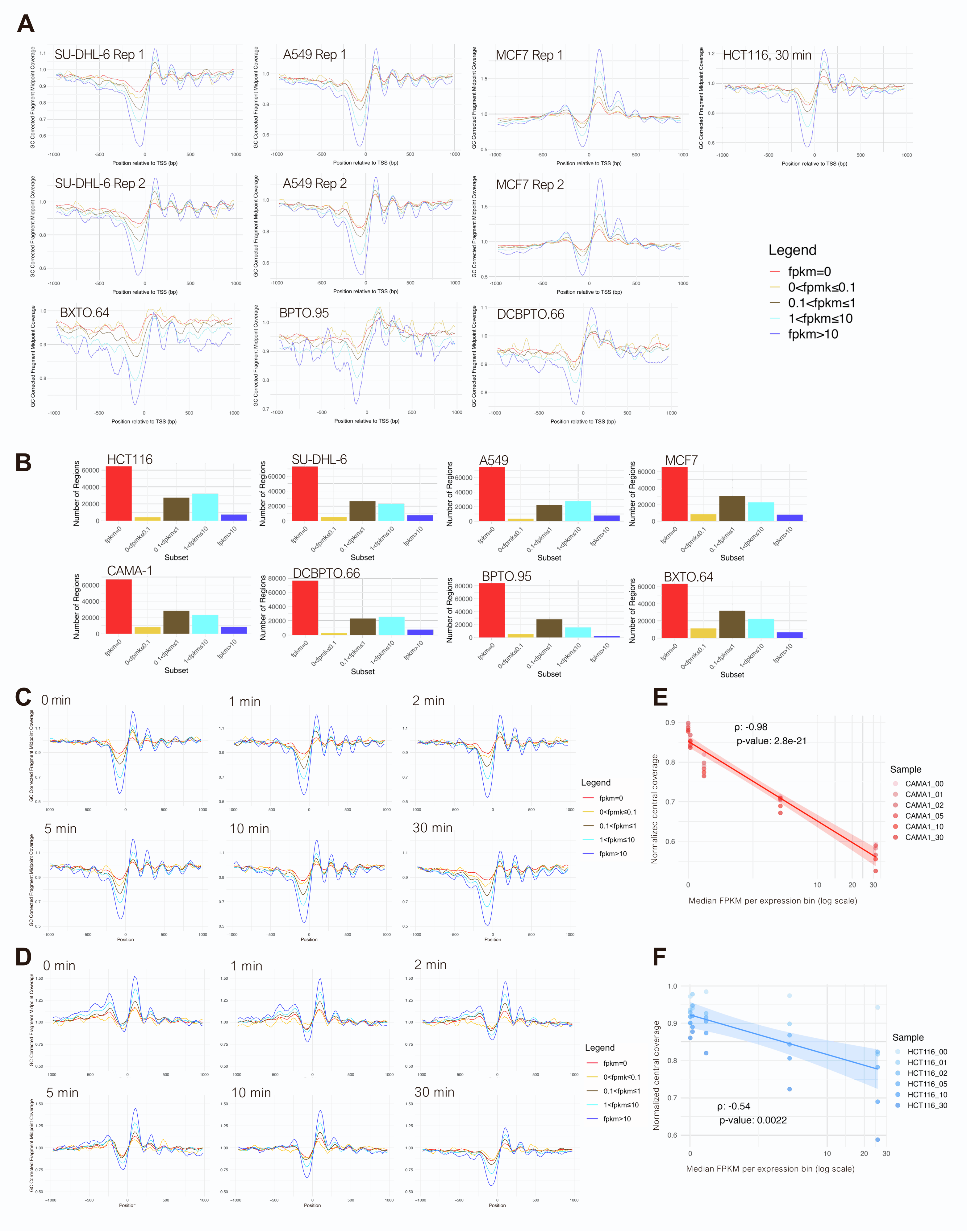
A
SU-DHL-6 Rep 1
A549 Rep 1
MCF7 Rep 1
HCT116, 30 min
SU-DHL-6 Rep 2
A549 Rep 2
MCF7 Rep 2
BXTO.64
BPTO.95
DCBPTO.66
GC Corrected Fragment Midpoint Coverage
Position relative to TSS (bp)
Legend
fpkm=0
0<fpmk≤0.1
0.1<fpkm≤1
1<fpkm≤10
fpkm>10
B
HCT116
SU-DHL-6
A549
MCF7
CAMA-1
DCBPTO.66
BPTO.95
BXTO.64
Number of Regions
Subset
fpkm=0
0<fpmk≤0.1
0.1<fpkm≤1
1<fpkm≤10
fpkm>10
C
0 min
1 min
2 min
5 min
10 min
30 min
Position
Legend
fpkm=0
0<fpmk≤0.1
0.1<fpkm≤1
1<fpkm≤10
fpkm>10
D
0 min
1 min
2 min
5 min
10 min
30 min
Position
Legend
fpkm=0
0<fpmk≤0.1
0.1<fpkm≤1
1<fpkm≤10
fpkm>10
E
ρ: -0.98
p-value: 2.8e-21
Normalized central coverage
Median FPKM per expression bin (log scale)
Sample
CAMA1_00
CAMA1_01
CAMA1_02
CAMA1_05
CAMA1_10
CAMA1_30
F
ρ: -0.54
p-value: 0.0022
Normalized central coverage
Median FPKM per expression bin (log scale)
Sample
HCT116_00
HCT116_01
HCT116_02
HCT116_05
HCT116_10
HCT116_30

Supplementary Figure 3, related to Figure 2. cfMNase-seq coverage is associated with gene expression around TSSs

(A) Composite cfMNase-seq coverage profiles at TSSs falling within five FPKM levels shown for all models (30-minute digestion). Coverage is shown as the average GC-corrected fragment midpoint coverage. (B) A bar graph representing the number of TSSs that fall into the five FPKM subsets (FPKM=0, 0<FPKM≤0.1, 0.1<FPKM≤1, 1<FPKM≤10, FPKM>10) for all models. (C) cfMNase-seq coverage profiles around the TSS for various gene expression levels and across MNase digestion times for CAMA-1. (D) cfMNase-seq coverage profiles around the TSS for various gene expression levels and across MNase digestion times for HCT116. (E) Normalized central coverage metrics from cfMNase-seq coverage profiles across different gene expression levels (Spearman correlation $\rho=-0.98$, $p=2.8\times10^{-21}$), shown for CAMA-1 samples of different digestion times. Normalized central coverage is the average normalized coverage between -125 and 25 bp centered around the TSS. (F) Normalized central coverage metrics from cfMNase-seq coverage profiles across different gene expression levels (Spearman correlation $\rho=-0.54$, $p=0.0022$), shown for HCT116 samples of different digestion times.

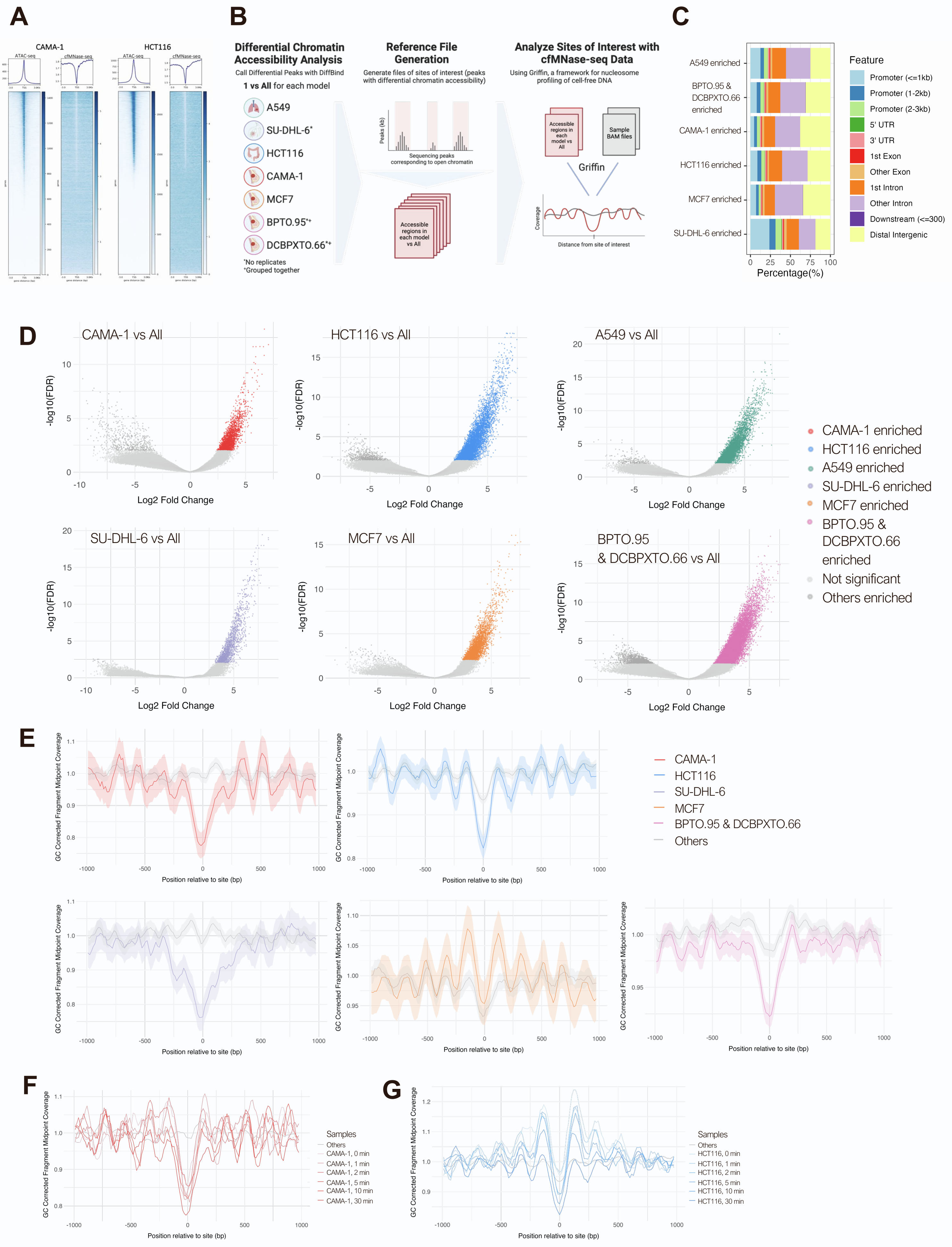

A
CAMA-1
HCT116
ATAC-seq
cfMNase-seq
B
Differential Chromatin Accessibility Analysis
Call Differential Peaks with DiffBind
1 vs All for each model
A549
SU-DHL-6*
HCT116
CAMA-1
MCF7
BPTO.95*+
DCBPXTO.66*+
*No replicates
+Grouped together
Reference File Generation
Generate files of sites of interest (peaks with differential chromatin accessibility)
Peaks (kb)
Sequencing peaks corresponding to open chromatin
Accessible regions in each model vs All
Analyze Sites of Interest with cfMNase-seq Data
Using Griffin, a framework for nucleosome profiling of cell-free DNA
Accessible regions in each model vs All
Sample BAM files
Griffin
Coverage
Distance from site of interest
C
A549 enriched
BPTO.95 & DCBPXTO.66 enriched
CAMA-1 enriched
HCT116 enriched
MCF7 enriched
SU-DHL-6 enriched
Percentage(%)
Feature
Promoter (<=1kb)
Promoter (1-2kb)
Promoter (2-3kb)
5' UTR
3' UTR
1st Exon
Other Exon
1st Intron
Other Intron
Downstream (<=300)
Distal Intergenic
D
CAMA-1 vs All
HCT116 vs All
A549 vs All
SU-DHL-6 vs All
MCF7 vs All
BPTO.95 & DCBPXTO.66 vs All
-log10(FDR)
Log2 Fold Change
CAMA-1 enriched
HCT116 enriched
A549 enriched
SU-DHL-6 enriched
MCF7 enriched
BPTO.95 & DCBPXTO.66 enriched
Not significant
Others enriched
E
GC Corrected Fragment Midpoint Coverage
Position relative to site (bp)
CAMA-1
HCT116
SU-DHL-6
MCF7
BPTO.95 & DCBPXTO.66
Others
F
Samples
Others
CAMA-1, 0 min
CAMA-1, 1 min
CAMA-1, 2 min
CAMA-1, 5 min
CAMA-1, 10 min
CAMA-1, 30 min
G
Samples
Others
HCT116, 0 min
HCT116, 1 min
HCT116, 2 min
HCT116, 5 min
HCT116, 10 min
HCT116, 30 min

Supplementary Figure 4, related to Figure 2. cfMNase-seq coverage is associated with chromatin accessibility

(A) Analysis of the association of chromatin accessibility and cfMNase-seq coverage around the TSS. Rows in the heatmap are individual genes, sorted by the ATAC-seq signal of the corresponding cell line. (B) Schematic of the differential chromatin accessibility analysis between models as regions of interest for cfMNase-seq coverage analysis. BPTO.95 and DCBPXTO.66 (xenograft version of DCBPTO.66) were grouped together for this analysis due to lack of replicates. Differential sites were identified with ATAC-seq peaks using EdgeR within diffbind. Sites with a log2 fold change greater than two and a false discovery rate less than 0.01 were counted as differential and were used to assess cfMNase-seq coverage. (C) Distribution of genomic features for sites with enriched accessibility for each model (CAMA-1, HCT116, SU-DHL-6, MCF7 and BPTO.95 and DCBPXTO.66) versus all others. Sites with a log2 fold change greater than two and a false discovery rate less than 0.01 were considered differential. (D) Volcano plots of differential sites considered for cfMNase-seq coverage analysis shown for each model versus all others. Sites with a log2 fold change greater than two and a false discovery rate less than 0.01 were considered differential and are shown in color; all others were not considered for cfMNase-seq coverage evaluation. (E) Composite cfMNase-seq coverage profiles (mean ± 95% CI across all sites) shown for 30-minute digestion samples at sites with enriched chromatin accessibility for CAMA-1 (2214 sites), HCT116 (6949 sites), SU-DHL-6 (1502 sites), MCF7 (3761 sites), and BPTO.95 and DCBPXTO.66 (18,202 sites). (F) Composite cfMNase-seq coverage profiles at 2214 sites with enriched chromatin accessibility for CAMA-1, shown for CAMA-1 across all digestion times and the average coverage across the other models. (G) Composite cfMNase-seq coverage profiles at 6949 sites with enriched chromatin accessibility for HCT116, shown for HCT116 across all digestion times and the average coverage across the other models.

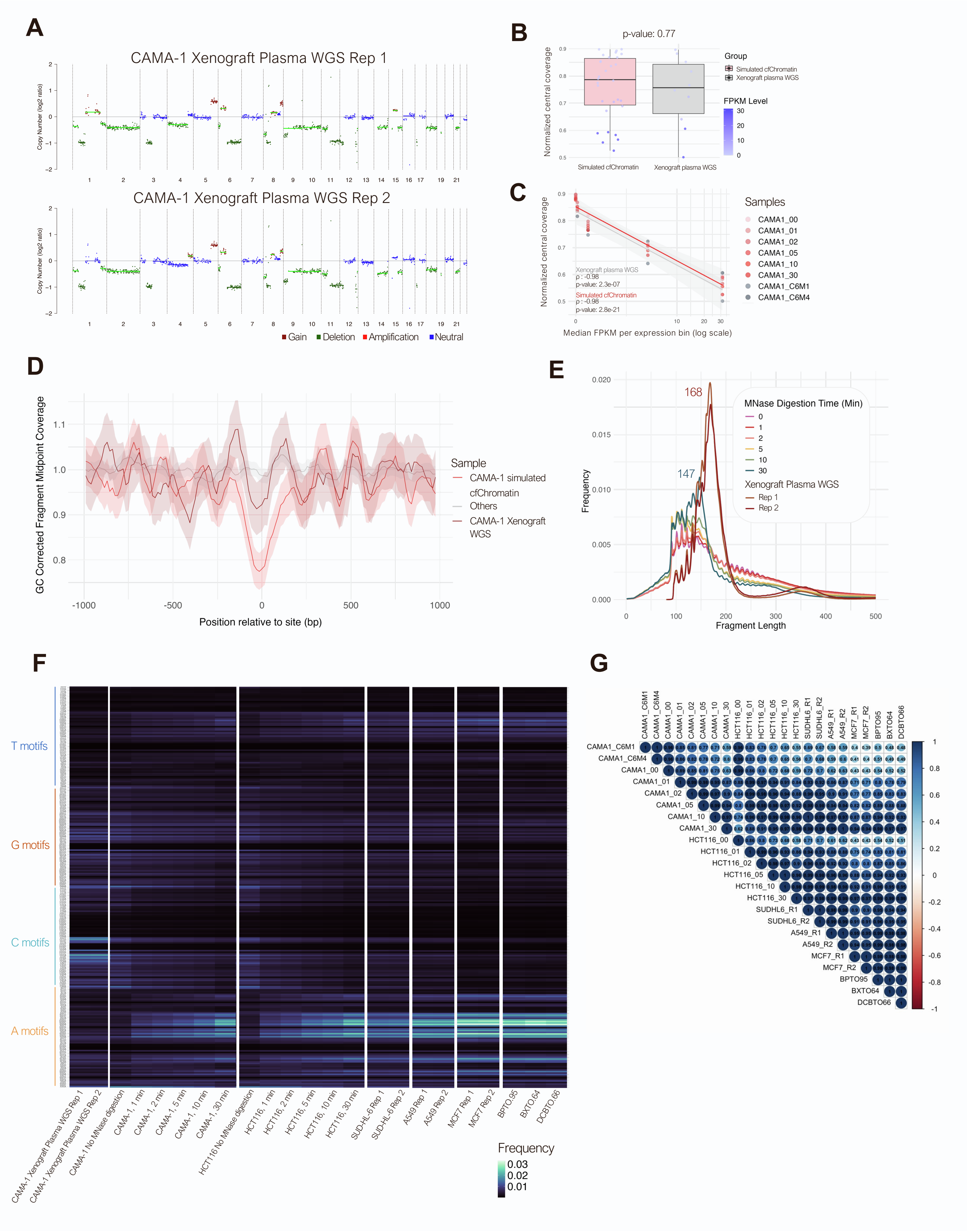
A
CAMA-1 Xenograft Plasma WGS Rep 1
CAMA-1 Xenograft Plasma WGS Rep 2
Copy Number (log2 ratio)
Gain
Deletion
Amplification
Neutral
B
p-value: 0.77
Normalized central coverage
Simulated cfChromatin
Xenograft plasma WGS
Group
FPKM Level
C
Normalized central coverage
Median FPKM per expression bin (log scale)
Xenograft plasma WGS
ρ : -0.98
p-value: 2.3e-07
Simulated cfChromatin
ρ : -0.98
p-value: 2.8e-21
Samples
CAMA1_00
CAMA1_01
CAMA1_02
CAMA1_05
CAMA1_10
CAMA1_30
CAMA1_C6M1
CAMA1_C6M4
D
GC Corrected Fragment Midpoint Coverage
Position relative to site (bp)
Sample
CAMA-1 simulated cfChromatin
Others
CAMA-1 Xenograft WGS
E
168
147
Frequency
Fragment Length
MNase Digestion Time (Min)
Xenograft Plasma WGS
Rep 1
Rep 2
F
T motifs
G motifs
C motifs
A motifs
CAMA-1 Xenograft Plasma WGS Rep 1
CAMA-1 Xenograft Plasma WGS Rep 2
CAMA-1 No MNase digestion
CAMA-1, 1 min
CAMA-1, 2 min
CAMA-1, 5 min
CAMA-1, 10 min
CAMA-1, 30 min
HCT116 No MNase digestion
HCT116, 1 min
HCT116, 2 min
HCT116, 5 min
HCT116, 10 min
HCT116, 30 min
SUD-HL-6 Rep 1
SUD-HL-6 Rep 2
A549 Rep 1
A549 Rep 2
MCF7 Rep 1
MCF7 Rep 2
BPTO.95
BXTO.64
DCBTO.66
Frequency
G
CAMA1_C6M1
CAMA1_C6M4
CAMA1_00
CAMA1_01
CAMA1_02
CAMA1_05
CAMA1_10
CAMA1_30
HCT116_00
HCT116_01
HCT116_02
HCT116_05
HCT116_10
HCT116_30
SUDHL6_R1
SUDHL6_R2
A549_R1
A549_R2
MCF7_R1
MCF7_R2
BPTO95
BXTO64
DCBTO66

Supplementary Figure 5, related to Figure 3. CAMA-1 cfMNase-seq chromosomal variation and nucleosome profiles are concordant with CAMA-1 xenograft plasma WGS whereas fragmentomic profiles differ

(A) Copy number analysis CAMA-1 xenograft plasma WGS samples. Corresponding CAMA-1 WGS and CAMA-1 cfMNase-seq copy number profiles can be found in Supplementary Fig. S2B and S2F. (B) Comparison of normalized central coverage metrics from coverage profiles across different gene expression levels for CAMA-1 cfMNase-seq (simulated cfChromatin) and CAMA-1 xenograft plasma WGS (Wilcoxon rank-sum test p=0.77). (C) Normalized central coverage metrics from coverage profiles across different gene expression levels for CAMA-1 cfMNase-seq samples (Spearman correlation ρ=-0.98, p=2.8×10^{-21}), and CAMA-1 xenograft plasma WGS samples (Spearman correlation ρ=-0.98, p= 2.3×10^{-07}). (D) Composite coverage profiles (mean ± 95% CI) at 2163 sites with enriched chromatin accessibility for CAMA-1, shown for CAMA-1 cfMNase-seq (30-minute digestion; central coverage: 0.78), CAMA-1 xenograft plasma WGS (merged replicates; central coverage: 0.92), and the the average of A549, BPTO.95, DCBPTO.66, HCT116 (30-minute digestion), MCF7, and SUD-HL-6 grouped together (central coverage: 0.99). (E) Fragment length distribution using picard collectInsertSize for CAMA-1 cfMNase-seq digestion time course and CAMA-1 xenograft plasma WGS samples. This demonstrates that cfMNase-seq samples exhibit a high frequency of fragments corresponding to the length of DNA wrapped around a nucleosome (147 bp). In contrast, xenograft plasma WGS samples show a higher frequency of fragments matching the length of DNA associated with a chromatosome (~166 bp). (F) Heatmap of 256 possible 5' end motif frequencies across all samples. Samples are arranged in groups by CAMA-1 xenograft plasma WGS, CAMA-1 time course, HCT116 time course, followed by all 30-minute digested cfMNase-seq samples. The heatmap demonstrates a decrease in C end motifs and increase in A end motifs as MNase digestion time increases. (G) Spearman correlation of 5' end motif frequencies across samples, displaying strong correlations between the xenograft plasma WGS and undigested samples cfChromatin samples, which decrease with increasing MNase digestion. CAMA1_C6M1=CAMA-1 Xenograft Plasma WGS Rep 1, CAMA1_C6M4=CAMA-1 Xenograft Plasma WGS Rep 2.

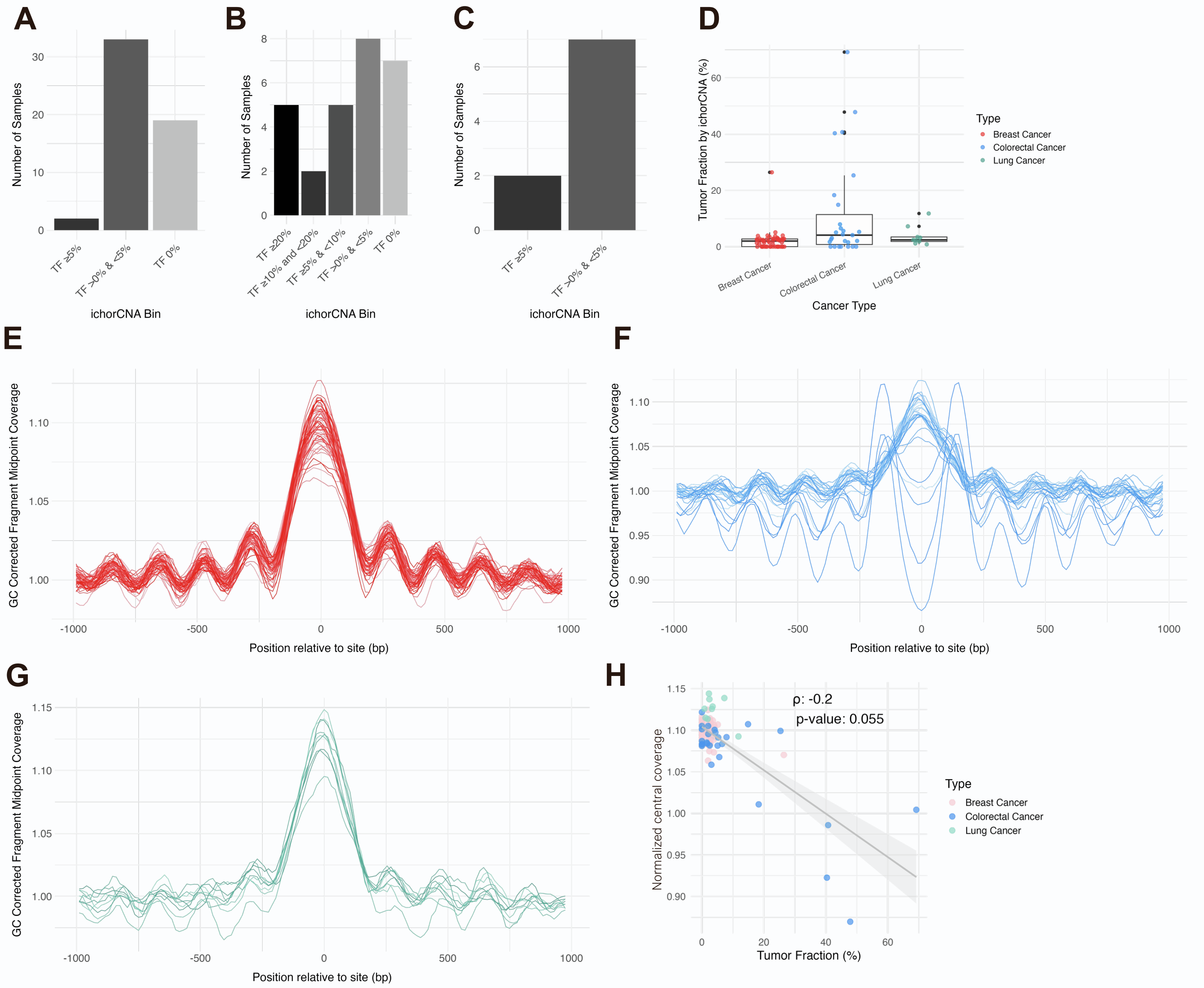

A
B
C
D
E
F
G
H
Number of Samples
ichorCNA Bin
TF ≥5%
TF >0% & <5%
TF 0%
TF ≥20%
TF ≥10% and <20%
TF ≥5% & <10%
Tumor Fraction by ichorCNA (%)
Cancer Type
Breast Cancer
Colorectal Cancer
Lung Cancer
Type
GC Corrected Fragment Midpoint Coverage
Position relative to site (bp)
Normalized central coverage
Tumor Fraction (%)
ρ: -0.2
p-value: 0.055

Supplementary Figure 6, related to Figure 3. Nucleosome positioning patterns of patient plasma WGS at open chromatin regions from patient tissue

(A) Numbers of breast cancer (n=54), (B) colorectal cancer (n=27), and (C) lung cancer patient plasma samples falling within ichorCNA bins. (D) Overview of plasma WGS samples tumor fractions for breast cancer (median=2.00, IQR=0-2.75), colorectal cancer (median=4.10, IQR=0.78-11.42), and lung cancer (median=2.42, IQR=1.86-3.46). (E) Composite coverage profiles at 117,917 ATAC-seq open chromatin sites from independent breast cancer patient samples within TCGA after filtering out PBMC signal shown for all breast cancer patient plasma WGS samples (n=54). (F) Composite coverage profiles at 56,484 ATAC-seq open chromatin sites from independent colon adenocarcinoma patient samples within TCGA after filtering out PBMC signal shown for all colorectal cancer patient plasma WGS samples (n=27). (G) Composite coverage profiles at 65,427 ATAC-seq open chromatin sites from independent lung adenocarcinoma patient samples within TCGA after filtering out PBMC signal shown for all lung adenocarcinoma patient plasma WGS samples (n=9). (H) Normalized central coverage metrics from nucleosome profiles at PBMC-filtered ATAC-seq open chromatin regions for each corresponding cancer type across different tumor fractions (Spearman correlation: ρ=-0.2, p=0.055).

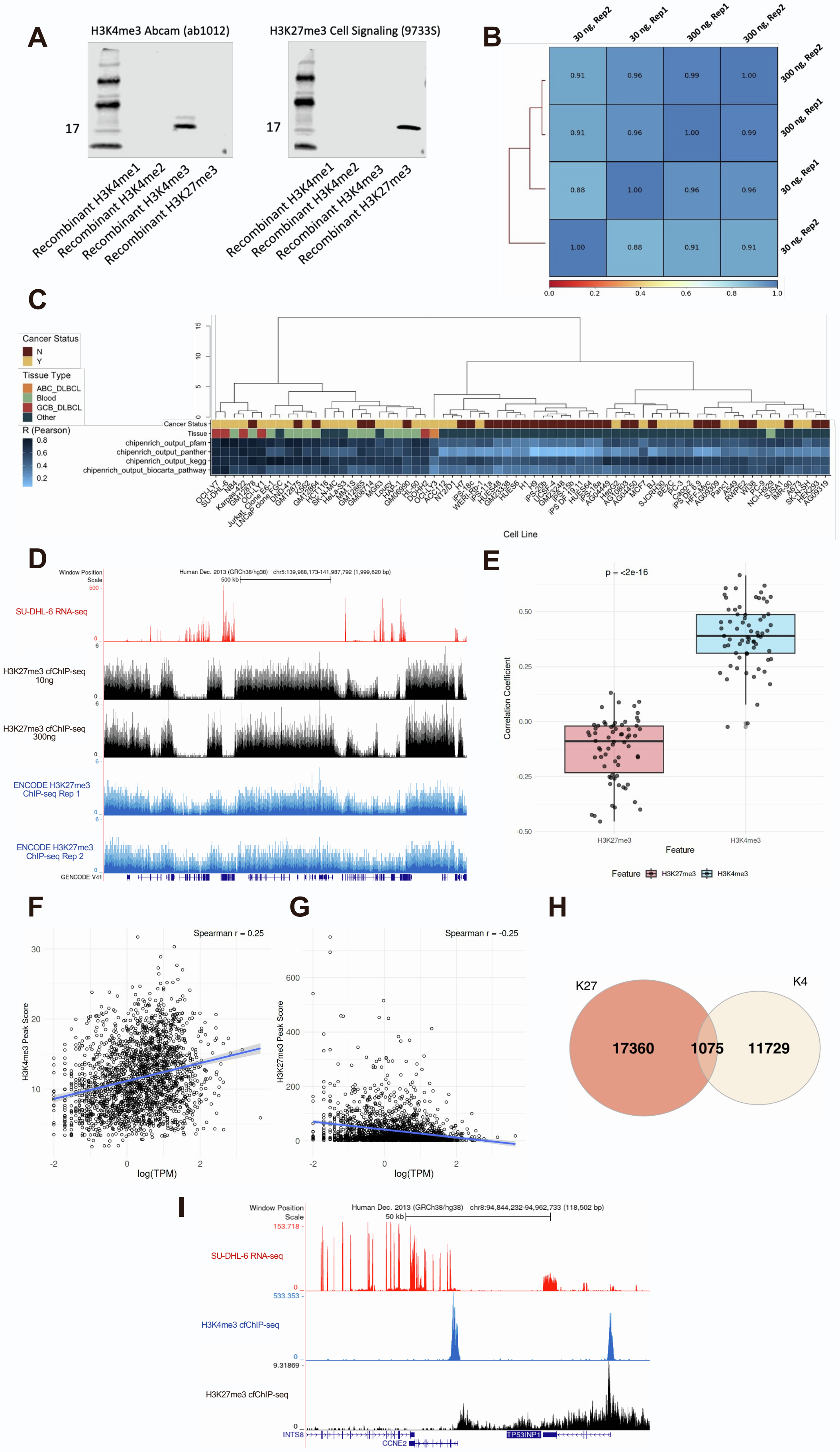

A
H3K4me3 Abcam (ab1012)
H3K27me3 Cell Signaling (9733S)
17
17
Recombinant H3K4me1
Recombinant H3K4me2
Recombinant H3K4me3
Recombinant H3K27me3
Recombinant H3K4me1
Recombinant H3K4me2
Recombinant H3K4me3
Recombinant H3K27me3
B
30 ng, Rep2
30 ng, Rep1
300 ng, Rep1
300 ng, Rep2
300 ng, Rep2
300 ng, Rep1
30 ng, Rep1
30 ng, Rep2
0.91 0.96 0.99 1.00
0.91 0.96 1.00 0.99
0.88 1.00 0.96 0.96
1.00 0.88 0.91 0.91
0.0 0.2 0.4 0.6 0.8 1.0
C
Cancer Status
N
Y
Tissue Type
ABC_DLBCL
Blood
GCB_DLBCL
Other
R (Pearson)
0.8
0.6
0.4
0.2
Cancer Status
Tissue
chipenrich_output_pfam
chipenrich_output_panther
chipenrich_output_kegg
chipenrich_output_biocarta_pathway
Cell Line
D
Window Position
Scale
Human Dec. 2013 (GRCh38/hg38) chr5:139,988,173-141,987,792 (1,999,620 bp)
500 kb
SU-DHL-6 RNA-seq
H3K27me3 cfChIP-seq 10ng
H3K27me3 cfChIP-seq 300ng
ENCODE H3K27me3 ChIP-seq Rep 1
ENCODE H3K27me3 ChIP-seq Rep 2
GENCODE V41
E
p = <2e-16
Correlation Coefficient
0.50
0.25
0.00
-0.25
-0.50
H3K27me3
H3K4me3
Feature
Feature H3K27me3 H3K4me3
F
Spearman r = 0.25
H3K4me3 Peak Score
log(TPM)
G
Spearman r = -0.25
H3K27me3 Peak Score
log(TPM)
H
K27
K4
17360
1075
11729
I
Window Position
Scale
Human Dec. 2013 (GRCh38/hg38) chr8:94,844,232-94,962,733 (118,502 bp)
50 kb
153.718
SU-DHL-6 RNA-seq
533.353
H3K4me3 cfChIP-seq
9.31869
H3K27me3 cfChIP-seq
INTS8
CCNE2
TP53INP1

Supplementary Figure 7, related to Figures 4-6. cfChIP-seq from SU-DHL-6 simulated cfChromatin reflects distinct chromatin states

(A) Preliminary validation of antibody specificity using recombinant histones (Active Motif) on a western blot. Antibodies against the desired target modifications (H3K4me3 and H3K27me3, respectively) were tested against recombinant H3K4me1, H3K4me2, H3K4me3, and H3K27me3, demonstrating antibody specificity. Additional data for the specificity of the H3K27me3 antibody (Cell Signaling) is described elsewhere[108]. (B) Pearson correlation across the coverage profiles from Figure 4B. Correlations were summarised over 1000 bp bins, genome wide. (C) Using ChIPEnrich, pathway analysis using peaks from SU-DHL-6 H3K4me3 cfChIP-seq profiles was performed and compared to ENCODE H3K4me3 profiles from various cell types (similar to Figure 4E). Each square in the heatmap represents a comparison between SU-DHL-6 H3K4me3 cfChIP-seq and H3K4me3 ChIP-seq from another cell line. Pearson correlation R values between odds ratios for particular pathway terms, for the different gene sets (including KEGG, Panther, Biocarta, and PFAM), are shown. Multiple gene sets were used to demonstrate correlations independent of the gene set. (D) Low and high input cfChIP-seq for H3K27me3 from SU-DHL-6 simulated cfChromatin shown, alongside replicates of SU-DHL-6 H3K27me3 ChIP-seq from ENCODE[69] and SU-DHL-6 RNA-seq[44]. BigWig files were RPKM normalized before visualization with the UCSC genome browser[48,109]. Tracks were visualized over a large genomic window on chromosome 5. (E) Boxplot of Spearman correlations between RNA expression and H3K4me3 (at TSSs) and H3K27me3 (at gene bodies), respectively, across 56 cell types from the REMC database and 9 cell types from ENCODE. (F) Spearman correlation between SU-DHL-6 media RNA expression and H3K4me3 expression at TSSs (ρ=-0.251, p<2.2e-16). (G) Spearman correlation between SU-DHL-6 media RNA expression and H3K27me3 expression at gene bodies (ρ=-0.253, p<2.2e-16). (H) Overlap of H3K4me3 and H3K27me3 MACS2 peaks from SU-DHL-6 simulated cfChromatin (both generated with 300 ng simulated cfChromatin as input). Overlapping peaks represent bivalent domains. (I) Visualization of an example bivalent promoter (TP53INP1) using the UCSC genome browser. Tracks represent RNA-seq from SU-DHL-6, H3K4me3 cfChIP-seq (300 ng input), and simulated cfChromatin H3K27me3 (300 ng input), respectively. All profiles were RPKM normalized before visualization.

Supplementary Table 1, Related to STAR Methods. Culture details

Culture medias used for cell lines in addition to supplementation with 10% FBS and 1% penicillin-streptomycin solution.

Cell line	Media
CAMA-1	Dulbecco's Modified Eagle Medium
HCT116	Roswell Park Memorial Institute 1640
CAMA-1	Dulbecco's Modified Eagle Medium
HCT116	Roswell Park Memorial Institute 1640
A549	Dulbecco's Modified Eagle Medium

Supplementary Table 2, Related to STAR Methods. Primer sequences

RNA primer sequences used for qPCR quantification of short human LINE-1 and ATAC-Seq quality control qPCR for enrichment of open regions (GAPDH and KAT6B) relative to closed regions (SLC22A3 and QML_93).

Target	Orientation	Primer Sequence
short human LINE-1	Forward	5'-TCACTCAAAGCCGCTCAACTAC-3'
short human LINE-1	Reverse	5'-TCTGCCTTCATTTCGTTATGTACC-3'
GAPDH	Forward	5'-GCC AAT CTC AGT CCC TTC CC-3'
GAPDH	Reverse	5'-TAG TAG CCG GGC CCT ACT TT-3'
KAT6B	Forward	5'-GAA GAG GCG GAC CCA GCG GT-3'
KAT6B	Reverse	5'-TTC CTG CCG GTC ATC TCG CTT-3'
SLC22A3	Forward	5'-GGA GAG GGT GGA CAG ATT GA-3'
SLC22A3	Reverse	5'-TCA GCC TTG CTG CTA CAG TG-3'
QML_93	Forward	5'-CAC TGG TTG TCT TTG CAG GA-3'
QML_93	Reverse	5'-CCT GGG TCA TAT TGG GAC AC-3'

Supplementary Table 3, Related to STAR Methods. Sample sequencing overview

Sample	Sequencer (Illumina)	Sequence length (bp)	Total Sequences
HCT116_noMNase	NovaSeq 6000	101	69163814
HCT116_01min_cfMNase	NovaSeq 6000	101	52810081
HCT116_02min_cfMNase	NovaSeq 6000	101	58723750
HCT116_05min_cfMNase	NovaSeq 6000	101	44856035
HCT116_10min_cfMNase	NovaSeq 6000	101	41224133
HCT116_30min_cfMNase	NovaSeq 6000	101	59539216
CAMA1_noMNase	NovaSeq 6000	101	51384474
CAMA1_01min_cfMNase	NovaSeq 6000	101	48978942
CAMA1_02min_cfMNase	NovaSeq 6000	101	47808513
CAMA1_05min_cfMNase	NovaSeq 6000	101	50138390
CAMA1_10min_cfMNase	NovaSeq 6000	101	48989715
CAMA1_30min_cfMNase	NovaSeq 6000	101	55385784
BXTO64_cfMNase	NovaSeq 6000	151	144651217
BPTO95_cfMNase	NovaSeq 6000	151	154850857
DCBPTO66_cfMNase	NovaSeq 6000	151	153984747
MCF7_cfMNase_Rep1	NovaSeq 6000	151	154024944
MCF7_cfMNase_Rep2	NovaSeq 6000	151	137741936
A549_cfMNase_Rep1	NextSeq 2000	101	103293962
A549_cfMNase_Rep2	NextSeq 2000	101	92245703
SUDHL6_cfMNase_Rep1	NextSeq 2000	101	72879151
SUDHL6_cfMNase_Rep2	NextSeq 2000	101	64288644
CAMA1_ATAC_Rep1	NovaSeq 6000	101	90401750
CAMA1_ATAC_Rep2	NovaSeq 6000	101	103873692
DCBXTO66_ATAC	NovaSeq 6000	51	63130776
BPTO95_ATAC	NovaSeq 6000	51	26687104
CAMA1_xenograft_plasma_Rep1	NextSeq 2000	101	92119733
CAMA1_xenograft_plasma_Rep2	NextSeq 2000	101	95512453
SUDHL6_H3K4me3_30ng_cfChIP_Rep1	NovaSeq 6000	100	10279688
SUDHL6_H3K4me3_30ng_cfChIP_Rep2	NovaSeq 6000	100	12384219
SUDHL6_H3K4me3_300ng_cfChIP_Rep1	NovaSeq 6000	100	8446289
SUDHL6_H3K4me3_300ng_cfChIP_Rep2	NovaSeq 6000	100	8041425
SUDHL6_H3K27me3_10ng_cfChIP_Rep1	NovaSeq 6000	101	163590521
SUDHL6_H3K27me3_10ng_cfChIP_Rep2	NovaSeq 6000	101	138894543
SUDHL6_H3K27me3_30ng_cfChIP_Rep1	NovaSeq 6000	101	135293892
SUDHL6_H3K27me3_30ng_cfChIP_Rep2	NovaSeq 6000	101	122040712
SUDHL6_H3K27me3_300ng_cfChIP_Rep1	NovaSeq 6000	101	107802286
SUDHL6_H3K27me3_300ng_cfChIP_Rep2	NovaSeq 6000	101	110221339
CAMA1_RNAseq	NovaSeq 6000	150	53598233
MCF7_RNAseq	NovaSeq 6000	150	48642926
DCBPTO66_RNAseq	NovaSeq 6000	150	32290393
BXTO_64_RNAseq	NovaSeq 6000	101	75840297
BPTO_95_RNAseq	NovaSeq 6000	101	66448731

Supplementary Table 4, Related to STAR Methods. 56 cell types and tissues used in this study from the REMC database

Source	ID	Cell Type/Tissue (Epigenome)
REMC	E003	H1_Cell Line
REMC	E004	H1_BMP4_Derived_Mesendoderm_Cultured_Cells
REMC	E005	H1_BMP4_Derived_Trophoblast_Cultured_Cells
REMC	E006	H1_Derived_Mesenchymal_Stem_Cells
REMC	E007	H1_Derived_Neuronal_Progenitor-Cultured_Cells
REMC	E011	hESC_Derived_CD184+_Endoderm_Cultured_Cells
REMC	E012	hESC_Derived_CD56+_Ectoderm_Cultured_Cells
REMC	E013	hESC_Derived_CD56-_Mesoderm_Cultured_Cells
REMC	E016	HUES64_Cell Line
REMC	E024	4star
REMC	E027	Breast_Myoepithelial_Cells
REMC	E028	Breast_vHMEC
REMC	E037	CD4_Memory_Primary_Cells
REMC	E038	CD4_Naive_Primary_Cells
REMC	E047	CD8_Naive_Primary-Cells
REMC	E050	Mobilized_CD34_Primary_Cells_Female
REMC	E053	Neurosphere_Cultured_Cells_Cortex_Derived
REMC	B054	Neurosphere_Cultured_Cells_Ganglionic_Eminence_Derived
REMC	E055	Penis_Foreskin_Fibroblast_Primary_Cells_skin01
REMC	B056	Penis_Foreskin_Fibroblast_Primary_Cells_skin02
REMC	E057	Penis_Foreskin_Keratinocyte_Primary_Cells_skin02
REMC	E058	Penis_Foreskin_Keratinocyte_Primary_Cells_skin03
REMC	E059	Penis_Foreskin_Melanocyte_Primary_Cells_skin01
REMC	BO61	Penis_Foreskin_Melanocyte_Primary_Cells_skin03
REMC	E062	Peripheral_Blood_Mononuclear_Primary_Cells
REMC	E065	Aorta
REMC	E066	Adult Liver
REMC	E070	Brain_Germinal_Matrix
REMC	E071	Brain_Hippocampus_Middle
REMC	E079	Esophagus
REMC	E082	Fetal_Brain_Female
REMC	BO84	Fetal_Intestine_Large
REMC	E085	Fetal Intestine_Small
REMC	E087	Pancreatic_Islets
REMC	E094	Gastric
REMC	E095	Left_Ventricle
REMC	E096	Lung
REMC	E097	Ovary
REMC	E098	Pancreas
REMC	E100	Psoas_Muscle
REMC	E104	Right_Atrium
REMC	E105	Right_Ventricle
REMC	E106	Sigmoid_Colon
REMC	E109	Small_Intestine
REMC	E112	Thymus
REMC	E113	Spleen
REMC	E114	A549
REMC	E116	GM12878
REMC	E117	HELA
REMC	D118	HEPG2
REMC	E119	HMEC
REMC	E120	HSMM
REMC	E122	HUVEC
REMC	E123	K562
REMC	E127	NHEK
REMC	E128	NHLE

Supplementary Table 5, Related to STAR Methods. H3K4me3 and H3K27me3 ENCODE data

H3K4me3 and H3K27me3 profiles from 68 cell lines used in this study, from the ENCODE database. If comma separated, samples indicate broad and narrow peaks, respectively. If accession ID is enclosed by square brackets, further processing of replicate BAM files was performed to broad and narrow peak file format.

Source	H3K4me3 Experiment ID	H3K27me3 Experiment ID(s)	Cell Line
ENCODE	ENCFF361TGO		A549
ENCODE	ENCFF272QER		A673
ENCODE	ENCFF081HTX		ACC112
ENCODE	ENCFF824TEY		AG04449
ENCODE	ENCFF988TOJ		AG04450
ENCODE	ENCFF949SYV		AG09309
ENCODE	ENCFF643VVG		AG09319
ENCODE	ENCFF313CEA		AG10803
ENCODE	ENCFF251CDW		BE2C
ENCODE	ENCFF364DKX		BJ
ENCODE	ENCFF642BGI<br>ENCFF001WWT,ENCFF876QHF	ENCFF001WWL,ENCFF604KXO	Caco-2
ENCODE	ENCFF069RHJ		DND-41
ENCODE	ENCFF375YDP		DOHH2
ENCODE	ENCFF253XQQ		GM06990
ENCODE	ENCFF800MHH		GM08714
ENCODE	ENCFF704LTU		GM12864
ENCODE	ENCFF438JMP		GM12865
ENCODE	ENCFF205BPF		GM12875
ENCODE	ENCFF320OGZ<br>ENCFF001WYJ,ENCFF795URC	ENCFF001WYB,ENCFF247VUO	GM12878
ENCODE	ENCFF123ETI		GM23248
ENCODE	ENCFF387WKX		GM23338
ENCODE	ENCFF041HYH<br>ENCFF001SVC,ENCFF192QQV	ENCFF001SUY,ENCFF434CYZ	H1
ENCODE	ENCFF985GWM<br>ENCFF001XAR,ENCFF207YLR	ENCFF001WZT,ENCFF302BSC	H7
ENCODE	ENCFF473AUA		H9
ENCODE	ENCFF856YLE		HAP-1
ENCODE	ENCFF187LLD		HCT116
ENCODE	ENCFF617NUV		HEK293
ENCODE	ENCFF469OXD		HeLa-S3
ENCODE	ENCFF549DKP<br>ENCFF001XDB,ENCFF712HMU	ENCFF001XCT,ENCFF042EDV	HepG2
ENCODE	ENCFF366OCN		HFF-Myc
ENCODE	ENCFF021JBH		HL-60
ENCODE	ENCFF441QBU		HUES48
ENCODE	ENCFF023OID		HUES6
ENCODE	ENCFF187LIH		HUES64
ENCODE	ENCFF093NQC		IMR-90
ENCODE	ENCFF450VVJ		iPS DF 19.11
ENCODE	ENCFF150NDM		iPS DF 6.9
ENCODE	ENCFF454SUG		iPS-11a
ENCODE	ENCFF724ZXV		iPS-15b
ENCODE	ENCFF618IAG		iPS-18a
ENCODE	ENCFF699VZO		iPS-18c
ENCODE	ENCFF483YLD		iPS-20b
ENCODE	ENCFF221IPH		Jurkat, Clone E6-1

Supplementary Table 5, Related to STAR Methods. H3K4me3 and H3K27me3 ENCODE data (continued)

H3K4me3 and H3K27me3 profiles from 68 cell lines used in this study, from the ENCODE database. If comma separated, samples indicate broad and narrow peaks, respectively. If accession ID is enclosed by square brackets, further processing of replicate BAM files was performed to broad and narrow peak file format.

Source	H3K4me3 Experiment ID	H3K27me3 Experiment ID(s)	Cell Line
ENCODE	ENCFF122CSI<br>ENCFF001XGT,ENCFF378OQB	ENCFF001SZF,ENCFF126QYP	K562
ENCODE	ENCFF693UVS<br>[ENCFF125FKI]	[ENCFF840XGT]	Karpas-422
ENCODE	ENCFF586HAM		LNCaP clone FGC
ENCODE	ENCFF329ZUR		Loucy
ENCODE	ENCFF145CCI		MCF7
ENCODE	ENCFF586UCO		MG63
ENCODE	ENCFF367SUW		MM.1S
ENCODE	ENCFF527YWO		NB4
ENCODE	ENCFF577HWW		NCI-H929
ENCODE	ENCFF954FXD		NT2/D1
ENCODE	ENCFF670JNI		OCI-LY1
ENCODE	ENCFF826PSG<br>[ENCFF224FBU]	[ENCFF254CXL,ENCFF375IRO]	OCI-LY3
ENCODE	ENCFF392YEW		OCI-LY7
ENCODE	ENCFF103AWU		Panc1
ENCODE	ENCFF375RGR		PC-3
ENCODE	ENCFF347EMU		PC-9
ENCODE	ENCFF965NTW		RWPE2
ENCODE	ENCFF639GVZ		SJCRH30
ENCODE	ENCFF870NJQ		SJSA1
ENCODE	ENCFF337FTH		SK-N-MC
ENCODE	ENCFF682JYE		SK-N-SH
ENCODE	ENCFF884HJC<br>[ENCFF408JOT]	[ENCFF182RDK]	SU-DHL-6
ENCODE	ENCFF742FZZ		UCSF-4
ENCODE	ENCFF287NSO		WERI-Rb-1
ENCODE	ENCFF033PCY		WI38
